# Supplementary material for: NeuroSAFE robot-assisted laparoscopic prostatectomy versus standard robot-assisted laparoscopic prostatectomy for men with localised prostate cancer (NeuroSAFE PROOF): protocol for a randomised controlled feasibility study
Source: BMJ Open. 2019 Jun 11;9(6):e028132. doi: 10.1136/bmjopen-2018-028132 (PMC6575674; doi:10.1136/bmjopen-2018-028132)
Supplement: Author's manuscript [file bmjopen-2018-028132.draft_revisions.pdf]

BMJ Open

BMJ Open is committed to open peer review. As part of this commitment we make the peer review history of every article we publish publicly available.

When an article is published we post the peer reviewers' comments and the authors' responses online. We also post the versions of the paper that were used during peer review. These are the versions that the peer review comments apply to.

The versions of the paper that follow are the versions that were submitted during the peer review process. They are not the versions of record or the final published versions. They should not be cited or distributed as the published version of this manuscript.

BMJ Open is an open access journal and the full, final, typeset and author-corrected version of record of the manuscript is available on our site with no access controls, subscription charges or pay-per-view fees (http://bmjopen.bmj.com).

If you have any questions on BMJ Open's open peer review process please email info.bmjopen@bmj.com

BMJ Open

A feasibility study RCT to investigate NeuroSAFE Robot-Assisted Laparoscopic Prostatectomy (RALP) versus standard RALP for men with localized prostate cancer: a study protocol for NeuroSAFE PROOF.

Journal:	*BMJ Open*
Manuscript ID	bmjopen-2018-028132
Article Type:	Protocol
Date Submitted by the Author:	23-Nov-2018

Complete List of Authors:	Dinneen, Eoin; University College Hospital London, Department of Urology<br>Haider, Aiman; University College Hospital London<br>Allen, Clare; University College Hospital London, Department of Radiology<br>Freeman, Alex; University College Hospital London, Department of Histopathology<br>Briggs, Tim; University College Hospital London, Department of Urology<br>Nathan, Senthil; University College Hospital London, Department of Urology<br>Brew-Graves, Chris; Division of Surgery and Interventional Science, University College London<br>Grierson, Jack; University College London Medical School, Division of Surgery & Interventional Science;<br>Williams, Norman; University College London, UCL Division of Surgery & Interventional Science<br>Persad, Raj; North Bristol NHS Trust<br>Oakley, Neil; Sheffield Teaching Hospitals NHS Foundation Trust<br>Adshead, Jim; Lister Hospital, Department of Urology<br>Huland, Hartwig; Universitatsklinikum Hamburg Eppendorf Universitares Herzzentrum Hamburg GmbH, Department of Urology<br>Haese, Alexander; Universitatsklinikum Hamburg Eppendorf Universitares Herzzentrum Hamburg GmbH, Department of Urology<br>Shaw, Greg; University College Hospital London, Department of Urology

Keywords:	NeuroSAFE, nerve sparing, frozen section, robotic prostatectomy, prostate cancer

SCHOLARONE™
Manuscripts

A feasibility study RCT to investigate NeuroSAFE Robot-Assisted Laparoscopic Prostatectomy (RALP) versus standard RALP for men with localized prostate cancer: a study protocol for NeuroSAFE PROOF.

Eoin Dinneen[1], Aiman Haider[2], Clare Allen[1], Alex Freeman[2], Tim Briggs[1], Senthil Nathan[1], Chris Brew-Graves[3], Jack Grierson[3], Norman R Williams[3], Raj Persad[4], Neil Oakley[5], Jim Adshead[6], Hartwig Huland[7], Alexander Haese[7], Greg Shaw[1].

Author Affiliations

1. University College Hospital London, Department of Urology, Westmoreland Street Hospital, 16-18 Westmoreland Street, London, W1G 8PH.
2. University College Hospital London, Department of Histopathology, 235 Euston Road, London NW1 2BU.
3. Surgical & Interventional Trials Unit, University College London, Charles Bell House, 43-45 Foley Street, London, W1W 7JN.
4. North Bristol Hospitals Trust, Department of Urology, Southmead Hospital, Southmead Lane, Westbury-on-Trym, Bristol, BS10 5NB.
5. Sheffield Teaching Hospitals NHS Trust, Department of Urology, Royal Hallamshire Hospital, Glossop Road, S10 2JF.
6. East and North Hertfordshire NHS Trust, Department of Urology, The Lister Hospital, Coreys Mill Lane, Stevenage, SG1 4AB.
7. Martini Klinik, Department of Urology, University Hospital Eppendorf, Martini-strasse 52, 20246 Hamburg.

Corresponding Author

Mr Eoin Dinneen BA (Hons), MRCS.
University College Hospital London, Department of Urology, Westmoreland Street Hospital, 16-18 Westmoreland Street, London, W1G 8PH.

eoin.dinneen@nhs.net

Word Count: 4,783.

Author email addresses (in the order they appear on authorship list):

aiman.haider@nhs.net, clare.allen@uclh.nhs.uk, alex.freeman2@nhs.net, tim.briggs@nhs.net, s.nathan@nhs.net, c.brew-graves@ucl.ac.uk, j.grierson@ucl.ac.uk, norman.williams@ucl.ac.uk, Raj.Persad@nbt.nhs.uk, neiloakley@btinternet.com, j.adshead@nhs.net, h.huland@uke.de, haese@uke.de, gregshaw@nhs.net.

Keywords

For peer review only

NeuroSAFE, nerve sparing, frozen section, potency, robotic prostatectomy, prostate cancer.

Abstract

Introduction

Robot assisted laparoscopic prostatectomy (RALP) is an effective cure for localized prostate cancer, but it is associated with considerable toxicity. Potency and urinary continence are improved when the neurovascular bundles (NVB) are spared during a nerve spare (NS) RALP. There is reluctance, however, to do NS RALP when there are concerns that the cancer extends beyond the capsule of the prostate into the NVBs, as NS RALP in this instance increases the risk of a positive surgical margin (PSM). The NeuroSAFE technique involves intraoperative fresh frozen section analysis of the postero-lateral aspect of the prostate margin to assess whether cancer extends beyond the capsule. There is evidence from large observational studies that functional outcomes can be improved and PSMs reduced when the NeuroSAFE technique is used alongside RALP. To date, however, there has been no randomised trial to substantiate this finding.

Methods

The NeuroSAFE PROOF trial is designed to assess whether it is feasible for men to be randomised to NeuroSAFE RALP against a control arm of 'standard of practice' RALP. NeuroSAFE PROOF will be a multicentre, single blinded feasibility RCT with patients randomised 1:1 to either NeuroSAFE RALP (intervention) or standard RALP (control). Treatment allocation will occur after trial entry and consent. The primary outcome will be assessed as the successful accrual of 50 men at 3 sites over 15 months. Secondary outcomes will be used to perform power calculations for the definitive larger-scale RCT and will include numbers of nerve spared PSMs, biochemical recurrence, adjuvant treatments , and functional outcomes reported by self-completion questionnaires on potency, continence and quality of life.

Ethics and dissemination

NeuroSAFE PROOF has ethical approval (REC reference 17/LO/1978). NeuroSAFE PROOF is supported NIHR Research for Patient Benefit funding (NIHR reference PB-PG-1216-20013). Findings will be made available to through peer-reviewed publications.

Trial Registration number

NCT03317990

For peer review only

Strengths and Limitations of this study

- This is the first feasibility clinical trial to compare NeuroSAFE RALP to a UK 'standard of care' RALP.
- Robust multicentre randomised controlled trial design.
- Feasibility study not powered for a definitive study.
- Surgical practices may differ systematically. This may influence outcome analysis.
- Secondary outcomes include validated patient reported outcome questionnaires, histological and oncological endpoints, and health economics.

Introduction

Nerve sparing (NS) robot-assisted laparoscopic radical prostatectomy (RALP) is associated with superior post-operative functional outcomes such as erectile function and possibly urinary continence. (1, 2) While functional results after RP are of importance to many men, the primary objective of an cancer operation remains complete eradication of the tumour.(3) Therefore, it is important performing NS RALP does not compromise that oncologic outcome. Positive surgical margins (PSM) are associated with greater risk of biochemical recurrence (4), adjuvant therapies (which negate any improved functional outcomes following NS RALP), and disease progression. As such, despite the improved anatomical understanding and technological advancement of the robotic platform, NS RALP has often been eschewed in favour of assuring the safety of a negative surgical margin. Uncertainty in this area is compounded by the fact that the accuracy of pre-operative imaging techniques and physical examination to detect extra-capsular extension and/or neurovascular cancer involvement are debatable and could lead to unwarranted sacrificing of important functioning nerves.(5, 6) Surgeons will therefore often rely on parameters such as pre-operative erectile function, biopsy Gleason score, radiological staging, and location and volume of tumour to cautiously assess the safety of an NS approach. These assessments may not give a true picture and are prone to subjective evaluation. The concept of a frozen section-navigated NS during RALP using neurovascular structure adjacent frozen section examination of the prostate resection margin (NeuroSAFE) has been described by the Martini-Klinik in Hamburg, Germany. (6-8) These authors and others report benefit in functional outcomes and improved oncologic safety in their series (9, 10) though other retrospective series are not as clear-cut.(11)

The NeuroSAFE technique has not yet been widely adopted, as concerns remain that it is time and resource consuming, has low sensitivity and specificity, and has potentially conflicting oncologic results.(12-15) Neither intraoperative fresh frozen section (FFS) in RALP nor the NeuroSAFE technique has been prospectively evaluated by an RCT. Moreover, few studies have assessed the impact of FFS during RALP on patient outcomes such as biochemical recurrence, adjuvant cancer treatments (such as radiotherapy and hormones), and comprehensive functional outcomes.

For peer review only

Research need

To determine whether the NeuroSAFE technique (fresh frozen section of the prostate tissue adjacent to the neurovascular bundles) during RARP is helpful to surgical teams (and therefore patients) who are balancing the competing goals of cancer control and functional optimization.(16) An attempt to answer this question will require a multi-dimensional approach focusing on pre-operative and operative parameters, final histological outcomes, adjuvant treatments, quality of life, erectile function, urinary continence and health economics. There is recognition that surgical RCTs can be hard to recruit to and that patients may not accept their allocated treatment option.(17) This is why we are undertaking this feasibility study to look at rates of recruitment, acceptance of allocated treatment and collection of outcomes.

Study aims and outcomes

The aim is to prospectively recruit for randomisation eligible patients to either standard RALP (control arm) or NeuroSAFE RALP (intervention arm). This feasibility trial has a single blinded, 1:1 randomised design. This article reports the protocol (v.2.0, 6 February 2018) for the NeuroSAFE PROOF trial and follows SPIRIT (Standard Protocol Items: Recommendations for Interventional Trials) reporting guidelines.(18)

The trial objectives are to assess the feasibility and acceptability of:

- Recruiting men with localized prostate cancer to a randomized study NeuroSAFE and exploring rates of attrition.
- Data collection for the outcome measures.
- Estimating cost effectiveness in a definitive trial.
- Estimate treatment effects in order to perform accurate power calculations to guide recruitment targets for any future full RCT.
- The study's procedures, interventions and follow-up regimen among patients being treated with RALP.

The following criteria will have to be met to proceed to a full-scale trial:

- Recruitment of 50 men over 15 months from opening.
- Recruitment and performance of procedures (both intervention and control RALP) as per allocation at 3 pre-specified participating sites (UCLH, Bristol and Sheffield). At least 2 treatments (one intervention, one control) should be performed at each site.

For peer review only

- Any issues with trial design identified in the feasibility stage can be addressed.
- Good acceptability of the intervention among patients and their families as indicated in qualitative feedback and Public & Patient Involvement events.
- Positive feedback from clinical staff and patients about scheduling clinics.
- Acquisition of comprehensive patient reported outcomes measure including health economics questionnaires.

Public and Patient Involvement (PPI)

Patient feedback on the design of the study was obtained at two NeuroSAFE PROOF PPI sessions on 12 July 2018 and 20th September 2018. The second event was attended by many of the men who are participating in NeuroSAFE PROOF. The PPI events were supported by Macmillan Cancer (Charity no. 261017) and Orchid (Charity no. 1080540) respectively. Participants, patients and their families were asked specifically about the level of blinding, the burden of follow-up appointments, and priorities in their recovery from RALP. Following their feedback, NeuroSAFE PROOF now informs men following surgery of their NS status, though blinding to allocation status (intervention or control) is maintained. Furthermore, men expressed keen preference to know their treatment allocation once exiting the 12 months follow-up period, and this is now incorporated into trial protocol. Patient representatives sit on the trial steering committee for NeuroSAFE PROOF and have oversight of the management of the research and analysis. Patient representatives also sit on the panel that evaluates the administration of NIHR funding for the Research for Patient Benefit stream. On completion of NeuroSAFE PROOF, prostate cancer patient groups will be consulted again on amendments to the design of the full NeuroSAFE RCT. The results will be published following peer review, and anonymised data will be presented at national and international conferences.

Methods and Analysis

Trial Design

NeuroSAFE PROOF is a prospective, multicentre, feasibility RCT in patients undergoing RALP for localised prostate cancer. Eligible patients will be consented and randomised 1:1 to NeuroSAFE RALP (intervention) or standard RALP (control) after multidisciplinary team (MDT) review in National Health Service (NHS) urological cancer centres. It is not possible to blind the surgical team to the treatment received on the day of surgery, however researchers co-ordinating participant follow-up will not routinely be informed of patient treatment allocation. Participants are not informed of treatment received until completing 12 months follow-up and exiting the study, though they are informed of their ultimate nerve-spare status (i.e. no nerve spare, unilateral nerve spare, bilateral nerve spare). The primary outcome is feasibility of recruitment.

For peer review only

Secondary outcomes will assess ability to perform treatment as per allocation, oncological outcomes, complications, and functional outcomes by collection the following data:

- Number of nerves spared,.
- Rates of positive surgical margins,.
- Adjuvant therapies and Biochemical Recurrence.
- Patient reported outcome questionnaires assessing potency, urinary continence, and quality of life.
- Health economics.

These outcome measures will allow us to explore the feasibility and acceptability of delivering a large-scale multicentre RCT.

Trial Population

The trial has a recruitment target of 50 patients (Figure 1). Prior to entry, patients must be accurately staged (e.g. mpMRI prostate and cross-sectional imaging to assess for distant metastases (e.g. bone scan or whole body MRI)), within 3 months prior to randomization. Eligible patients must have had their case discussed at NHS cancer MDT and deemed suitable and fit for RALP. Eligible participants will fulfil all the inclusion criteria and none of the exclusion criteria as defined below:

Inclusion Criteria

i. Men opting to undergo RALP for organ confined prostate cancer.
ii. Potent men (IIEF 22-25 not using PDE51 or other medications or vacuum pump)
iii. Men who are continent of urine (no self-reported urinary incontinence)
iv. Able to give written informed consent to participate.

Exclusion Criteria

i. Unable to undergo RALP
ii. Known overactive bladder
iii. Previous treatment for prostate cancer
iv. Previous/current hormone treatment for prostate cancer
v. Nerve sparing deemed futile due to locally advanced disease by surgeon and radiologist.

Sample Size

The primary outcome of NeuroSAFE PROOF is to demonstrate adequate recruitment to prove feasibility of the large-scale definitive RCT. The primary outcome measures intended for the full RCT (oncological outcomes and

For peer review only

functional outcomes) will be determined by this trial. The rates of positive surgical margins and the NVBs spared in the two groups will be used together with data from previously published literature to inform power calculations for the full scale trial. Previous literature suggests that 80% of men undergoing bilateral NS will have erections sufficient for penetrative sex, 40% of men undergoing unilateral NS and 10% of men undergoing no NS. (19)

For peer review only

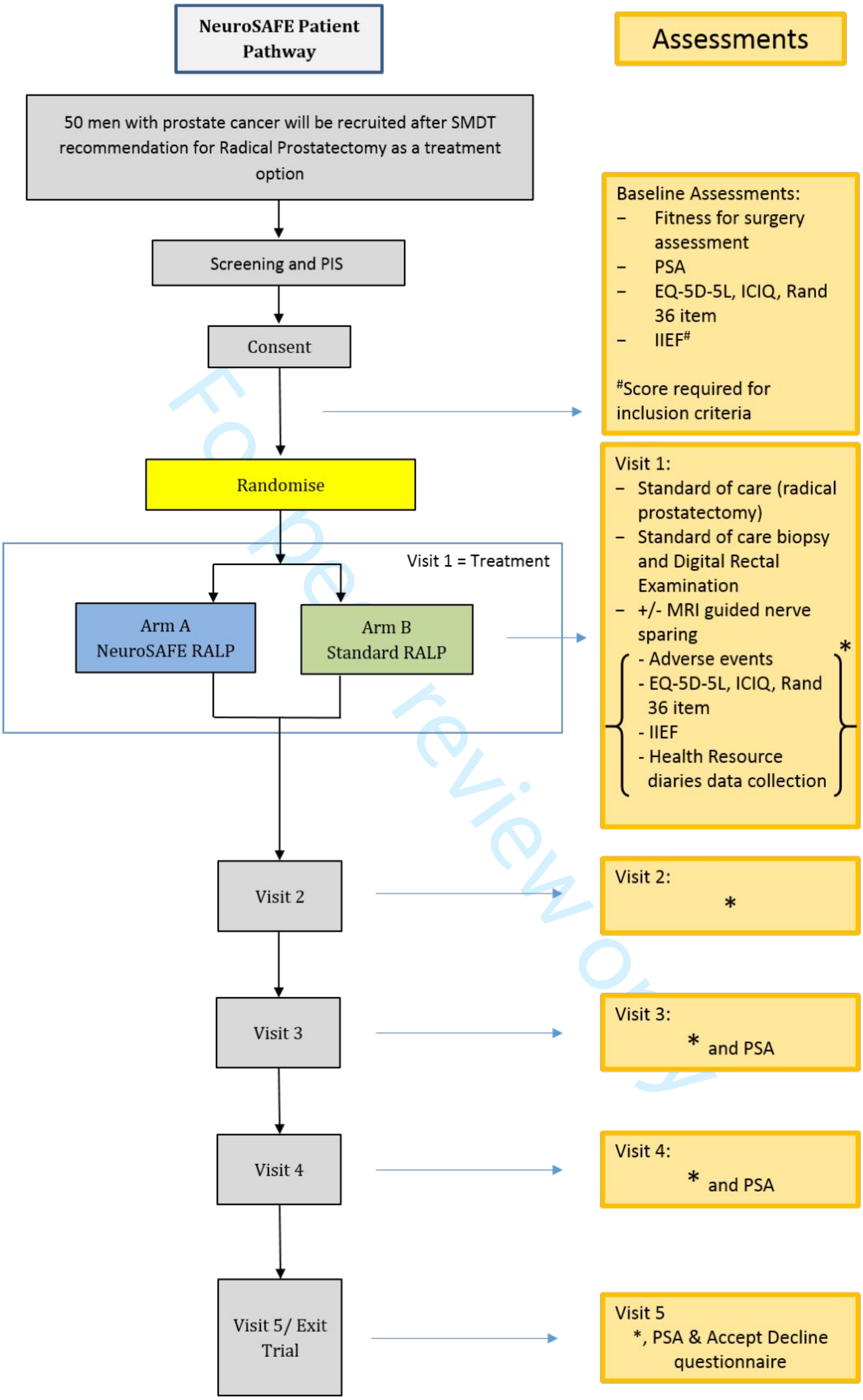

Figure 1. Study Flow Chart. NeuroSAFE PROOF.

For peer review only

Recruitment

NeuroSAFE PROOF will recruit patients attending NHS cancer centres. All patients who have a diagnosis of prostate cancer in whom the specialist MDT has recommended RALP as a treatment option can be approached.

Consent

Written informed consent will be obtained from each patient prior to study entry and performing baseline trial assessments. An ethics committee approved patient information sheet will be provided to facilitate this process. Prospective participants will be given at least a week to read the patient information sheet prior to being re-approached with regards to recruitment. The investigator, or their designee, must ensure adequate explanations of the trial that participation is voluntary, and they can withdraw at any time. In consenting to the trial participants understand that they are consenting to provide study follow-up and data collection. A patient may withdraw from the trial at any time without prejudice to his subsequent treatment.

Randomisation

Patients will be randomised using an online system (https://www.sealedenvelope.com/trials/) on a 1:1 basis to either NeuroSAFE RALP or standard RALP. A computer-generated adaptive minimization algorithm that incorporates a random element will be used to ensure treatment groups are balanced (stratified) for centre. Treatment allocation will occur after trial entry and consent.

Setting

Participants will be recruited from NHS cancer centres undertaking RALP who have the ability to perform the additional NeuroSAFE technique. Recruiting sites will be invited by the Trial Management Group (TMG) as having well-developed RALP programs with sufficient volume to recruit a reasonable number for patients to the trial.

Surgeon and unit accreditation

Variations in surgical team performance can produce differences in outcomes from RALP.(20) As such, to minimize this potential source of confounding, surgeons and surgical teams undertaking RALP within NeurosAFE PROOF require accreditation from the TMG. Further, surgeons performing trial treatment need to have completed more than 100 cases and have submitted these data to the BAUS Oncology database.

Robot-assisted Laparoscopic Prostatectomy (RALP)

Patients will undergo robot assisted radical laparoscopic prostatectomy using the DaVinci surgical system as is standard of care in the NHS. All patients will undergo a pre-operative multi-parametric MRI (mpMRI) that will be interpreted by a consultant genitourinary radiologist experienced in MRI. The pre-operative mpMRI will be interpreted by the radiologist with biopsy information and will be used to evaluate presence of cancer and likelihood of extra-capsular extension in zones according to the PIRADS anatomic division of the prostate at the base, the mid gland and the apex. In each zone, using a 1-5 scale (1, definitely absent; 2, probably absent; 3, possibly present; 4, probably present; 5 definitely present), they will record the likelihood of tumour on each side. Using the same 1-5 scale they then indicated the likelihood of extra-capsular extension in each corresponding zone as has been previously done by Akin et al.(21) Subsequently, the radiologist using the mpMRI makes a NS recommendation for each side of the prostate for each participant regardless of treatment arm allocation. The radiological NS recommendation will be recorded:

- Nerve Sparing: Yes
- Nerve Sparing: No
- Digital rectal examination dependent.

Control Arm: Standard RALP

Routine RALP is performed as per NHS standard practice. Pre-operative parameters used to guide surgeon NS decision include mpMRI review with genitourinary radiologist recommendation with regards NS, prostate biopsy histology, and digital rectal examination under general anaesthesia. Individual surgeons are asked after RALP to grade the quality of NS performed on each side numerically as seen below as previously described (22):

- Grade 4 - No nerve spare. Wide excision of lateral pelvic fascia (LPF) and Denonvilliers' fascia.
- Grade 3 - Limited nerve spare, or partial/incremental nerve spare. Incision through outer compartment of LPF.
- Grade 2 – Interfascial nerve spare. LPF is taken just outside the layer of the veins of the prostate capsule. Still largely preserving the large neural trunks.
- Grade 1 – Intrafascial nerve spare. LPF is taken just outside the prostate capsule. Represents greatest possible NS.

Detailed times of starting the RALP and finishing the RALP are recorded on the day of surgery in order to calculate the length of each case.

Intervention Arm: NeuroSAFE RALP

NeuroSAFE RALP has performed in accordance to previously described methods.(6, 8, 23) The additional steps outlined include nerve sparing technique and apical dissection, specimen removal, intra-operative frozen section protocol, simultaneous urethra-vesical anastomosis (+/- pelvic lymphadenectomy where

For peer review only - http://bmjopen.bmj.com/site/about/guidelines.xhtml

performed), pathological processing of specimen, pathology reporting protocol, and secondary excision of the neurovascular bundle (where appropriate). When the FFS analysis demonstrates cancer at the margin of the prostate as per pathology reporting protocol, secondary excision of the NVB is described by the surgeon in one of three ways: 1. No tissue resected, 2. Local excision of Denonvilliers'/peri-prostatic fascia, or 3. Entire bundle resected. Secondarily resected tissue (after fresh frozen section pathology phone call, when performed) is sent for routine paraffin embedded histological analysis and is not analysed as part of the intraoperative fresh frozen section. Detailed times of the beginning of the RALP, the removal of the prostate for specimen painting, arrival of specimen in laboratory, communication of details of fresh frozen section to the surgical team, and finishing the RALP are recorded on the day of surgery.

Participating sites all visited the central site (UCLH) prior to their Site Initiation Visits in order to receive teaching and standardisation in the surgical and histopathological aspects of NeuroSAFE RALP (intervention arm). Subsequently, researchers from the central site (GS and AH) reciprocated the visit for the first NeuroSAFE RALP performed by each site to ensure fidelity to technique protocol.

Data Collection

Trial Assessments will be conducted at carious time intervals (defined around the date of surgery). Schedule of events is summarized in Table 1. Time points:

i. Baseline/preoperative: at time of consent, trial entry and randomisation to treatment allocation.
ii. Visit 1. Operative parameters recorded and any immediate post-operative complications/adverse events.
iii. Outpatient follow-up. Visit 2 includes records patient reported outcome measures including health economics follow-up.
iv. Visits 3, 4 and 5 will record patient reported outcomes measure with the addition of PSA blood tests. Adjuvant treatments and oncological outcomes will be recorded prospectively alongside functional assessments.

For peer review only

Visit	Screening/Baseline	Randomisation	Treatment Visit 1	Visit 2 6 weeks post op (+ 4 weeks)	Visit 3 3 months (±4 weeks)	Visit 4 6 months (±4 weeks)	Visit 5 12 months (±4 weeks)
Informed consent	x						
Randomisation		x					
Fitness for surgery assessment	x						
PSA	x				x	x	x
Standard care (radical prostatectomy)			x				
Standard care biopsy & DRE			x				
+/-MRI guided nerve sparing			x				
Adverse events			x	x	x	x	x
EQ-5D-5L, ICIQ, Rand 36 items, ICIQ	x		x	x	x	x	x
IIEF	X[1]		x	x	x	x	x
Health Resource Diaries data collection			x	x	x	x	x
Accept Decline Questionnaire							x

[1] needs to be done prior to randomisation as score is required for inclusion criteria, all other baseline questionnaires, need to be completed prior to telling the patient which treatment arm he has drawn.

Table 1. Table of Assessments.

Secondary endpoint measures include

i. IIEF-15 (baseline, 6 weeks, 3 months, 6 months and 12 months): a self-completion tool for men focusing on erectile function and sex life. Measured domains include erectile function, orgasmic function, sexual desire, intercourse satisfaction and overall satisfaction.(24)

ii. Rand-36-item Health Survey (baseline, 6 weeks, 3 months, 6 months and 12 months): a self-completion questionnaire that laps eight concepts: physical functioning, bodily pain, role limitations due to health problems, role limitations due to personal or emotional problems, emotional well-being, social functioning, energy/fatigue, and general health perceptions.(25)

iii. International Consultation on Incontinence Questionnaire (ICIQ) (baseline, 6 weeks, 3 months, 6 months, and 12 months): a self-completion tool for patients to subjectively measure frequency and severity of urinary loss, and impact on quality of life for those with urinary incontinence.(26)

i. EQ-5D-5L (baseline, 6 weeks, 3 months, 6 months, and 12 months): a self-completion tool for patients that is applicable to a wide range of health conditions and treatments. Measured domains include mobility, self-care, usual activities, pain and anxiety or depression.(27)

ii. Health resource diaries (6 weeks, 3 months, 6 months, six month visit diary will be returned at the 12 month visit),. This will allow the collection of resource use data from point of operation untill trial exit at 12 months. These diaries are non-validated.

iii. Post-operative: adverse events and complications will be recorded. Clavien-Dindo classification of surgical complications will be used to assess for any surgical complications as per normal hospital practice.

iv. Histology: following RALP the following details will be recorded as per standard histological analysis of prostatectomy mount: histological

type, Gleason Grade, Gleason group, tumour volume, extra prostatic extension, seminal vesicle involvement, lymphovascular invasion, description of margin involvement (including apical, basal, circumferential), tumour stage, nodes, positive surgical margins.

v. Oncological outcomes (3 months, 6 months and 12 months): the curative outcomes from RALP will be examined to determine local and distant recurrence, metastases, PSA and biochemical recurrence, need for adjuvant therapies and survival (overall and cancer specific).

Statistical Analysis

As NeuroSAFE PROOF is a feasibility trial, detailed statistical analysis will not be undertaken of the primary outcome. Preliminary analysis will be performed after 5 cases have reached Visit 3 to rehearse data extraction, completeness of follow-up, fidelity of data, and by proxy acceptability of follow-up measures. Further preliminary statistical analysis, maintaining blinding, of the secondary outcomes (margin status and nerves spared) will be performed by the data monitoring committee (DMC) after 40 surgeries have been performed to evaluate and revise power calculations for the full-scale definitive RCT. Potential bias due to missing data will be investigated by comparing descriptively the baseline characteristics of the trial participants with complete outcome measurements to those who have missing outcome measurements. Men will be offered the option of telephone follow-up and/or be sent questionnaires by post if they are unable to attend clinic appointments for follow-up. Additionally, patients wishing to withdraw from the trial will be counselled regarding end of active participation, as this would allow the trial team to collect outcome data for an intention to treat (ITT) analysis. Records will be kept of all participants allocated to a treatment arm who do not undergo allocated treatment with explanatory notes. These instances will be highlighted to SITU and the TSC for judgment on whether inclusion in outcomes is appropriate.

Safety

The number of adverse events related to serious adverse events (SAEs) will be summarised descriptively by arm, by grade and body system. RALP is a major operation that has a number of recognized complications and a very low risk of death (less than 1 in 100). Operative/post-operative RALP complications will be graded using the Clavien-Dindo classification.(28) All SAEs will be recorded in the medical records, the CRF, the sponsor's adverse event log, and an SAE form. The principal investigator (PI) or designated individual will complete an SAE form, and he form will be sent to the surgical and interventional trials unit (SITU) within five working days of becoming aware of the event. The chief or PI will respond to any SAE queries raised by the sponsor as soon as possible. Where the event is unexpected and thought to be related to the procedure, this must be reported by the investigator to SITU, who will then inform the Health Research Authority within 15 days.

For peer review only - http://bmjopen.bmj.com/site/about/guidelines.xhtml

Data Monitoring

This trial will use an electronic case report form (eCRF), and trial data will be entered into an approved, protected database (https://neurosafe.slms.ucl.ac.uk). Access to the eCRF system will only be provided to staff with the appropriate authority. Participants will be given a unique number and subject identifier. Data will be entered under this identification number onto the central database stored on the servers. The database will be password protected and only accessible to members of the NeuroSAFE study team and external regulators if requested. The servers are protected by firewalls and are patched and maintained according to best practice. The physical location of the servers is protected by CCTV and security door access. The database software provides a number of features to help maintain data quality, including: maintaining an audit trail, allowing custom validations on all data, allowing users to raise data query requests and search facilities to identify validation failure/missing data. After completion of the study, the database will be retained on the servers of University College London for on-going analysis of secondary outcomes. The identification, screening and enrolment logs, linking participant identifiable data to the pseudo-anonymised subject numbers will be held in written form in a locked filing cabinet. After completion of the study, sites will store screening and enrolment logs securely for 10 years.

Trial Funding, organization and administration

The trial was developed by the NeuroSAFE PROOF TMG and has been funded by University College Hospital London Trust, Rosetrees Foundation, and the NIHR Research for Patient Benefit (RfPB) stream (reference: PB-PG-1216-200113). Applied Medical are contributing disposable laparoscopic trocar ports suitable for use in NeuroSAFE RALP (intervention arm) but has had no role in trial design and will have no role in trial implementation, analysis, interpretation or writing any reports. The trial is sponsored by University College London and has registered sponsor reference number 17/0443 and ClinicalTrials.gov (NCT03317990) on 23 October 2017 with an amendment made on 1 June 2018. All members of the trial are Good Clinical Practice trained. A Data Monitoring Committee (DMC) will monitor patient safety and the rate of recruitment of subjects in the study. They will meet at least once a year while the trial is ongoing for routine review of safety data and trial progression. They have power to call additional meetings and review data at any point in the trial should they wish to do so. The DMC may report their findings to the TSC. The TSC is an independent committee consisting of relevant, experienced clinicians and researchers. The TSC will ensure the study is conforming to governance requirements as set out by the trial sponsor. The TSC will meet at least once a year. The sponsor may also arrange an independent trial monitor to review the study data.

Ethics and Dissemination

Ethical approval for NeuroSAFE PROOF was granted on 6th February 2018 (REC reference 17/LO/1978). It is an NHS NIHR Research for Patient Benefit funded

For peer review only

study (NIHR reference PB-PG-1216-20013). The study is sponsored by University College London (Sponsor reference number: 17/0443). Here, we report version 2.0 of the protocol. The sponsors, HRA, and REC will approve any future amendments as appropriate. Similarly, all participating centres have gained local REC prior to receiving a site initiation visit and being given the permission to open recruitment.

Non-blinded results of the study will be published in peer-reviewed publications and will be presented at relevant national and international conferences. The TMG will not present the arms in comparison to one another to avoid loss of equipoise and introduction of bias into the full-scale RCT. The TMG will work with the patient panel to develop lay reports to disseminate research findings to patient groups and the clinical teams at participating sites.

Discussion

Intra-operative FFS analysis of the NVB adjacent prostate tissue during RALP to guide NS is now an established technique in a number of centres. Published large series from these centres demonstrate improvements on their PSM rates, oncological and functional outcomes. Conversely, other authors describe limited benefit in their experience. In spite of possible benefit to men undergoing RALP, and perhaps for health economic reasons NeuroSAFE and/or similar intraoperative FFS analyses at the time of NS RALP have had limited uptake in the UK. The lack of Level 1 evidence to support NeuroSAFE could be a valid reason why progress, which could be of great benefit to patients, has not been made in this area.

NeuroSAFE PROOF will be the first trial to assess the feasibility of conducting an RCT to evaluate fresh frozen section analysis as an intra-operative surgeon consultation to guide nerve spare surgery in RALP. The results of this feasibility trial will be used to decide whether to progress to a full-scale trial, and if so, what methodological issues may need to be addressed and changed.

Trial Status

NeuroSAFE PROOF opened to recruitment in April 2018 using protocol version 2.0 (6 February 2018) and is due to close to recruitment in January 2020 after the 50th patient is consented and randomised. NeuroSAFE PROOF will close in January 2021 after the last patient to undergo treatment in either RALP arm will have completed their final follow-up visit and exited the trial. Amendments were reviewed and approved by the sponsor and the Regional Ethics Committee. Protocol amendments are disseminated to relevant parties by SITU.

Acknowledgements

We gratefully thank the participants, principal investigators, research nurses, MDT coordinators, data managers and other site staff who have been responsible for setting up, recruiting participants and collecting the data for the trial. We are grateful for the trial oversight provided by the sponsor and the members of the

For peer review only

Trial Steering Committee. (TSC). The TSC members are Jack Cuzick (Chair), Queen Mary's University of London, Alastair Lamb, Oxford, Imran Ahmad, Glasgow, and Abay Mulatu, Coventry.

Contributors

Conception and design of NeuroSAFE PROOF trial: GS, ED, AH, JG, AF, CA, JA, RP, NO and CB-G. Writing of the manuscript: ED, AH, CA, AF, RP, NO, CB-G, JG, and GS. All authors have read and approved the final manuscript. The trial will comply with the authorship criteria recommended but the International Committee of Medical Journal Editors.

Funding

The trial was funded by the NIHR RfPB and The Rosetrees Foundation. ED is funded by the NIHR RfPB.

Disclaimer

The funder had no role in the design, analysis, or collection of the data; in writing the manuscript; or in the decision to submit the manuscript for publication.

Competing Interests

Within NeuroSAFE PROOF, laparoscopic ports are supplied by Applied Medical but Applied Medical has had no role in the design, analysis, or collection of the data; in writing the manuscript; or in the decision to submit the manuscript for publication

For peer review only

References

1. Steineck G, Bjartell A, Hugosson J, Axen E, Carlsson S, Stranne J, et al. Degree of preservation of the neurovascular bundles during radical prostatectomy and urinary continence 1 year after surgery. Eur Urol. 2015;67(3):559-68.
2. Savera AT, Kaul S, Badani K, Stark AT, Shah NL, Menon M. Robotic radical prostatectomy with the "Veil of Aphrodite" technique: histologic evidence of enhanced nerve sparing. Eur Urol. 2006;49(6):1065-73; discussion 73-4.
3. Ficarra V, Sooriakumaran P, Novara G, Schatloff O, Briganti A, Van der Poel H, et al. Systematic review of methods for reporting combined outcomes after radical prostatectomy and proposal of a novel system: the survival, continence, and potency (SCP) classification. Eur Urol. 2012;61(3):541-8.
4. Sooriakumaran P, Ploumidis A, Nyberg T, Olsson M, Akre O, Haendler L, et al. The impact of length and location of positive margins in predicting biochemical recurrence after robot-assisted radical prostatectomy with a minimum follow-up of 5 years. BJU Int. 2015;115(1):106-13.
5. de Rooij M, Hamoen EH, Witjes JA, Barentsz JO, Rovers MM. Accuracy of Magnetic Resonance Imaging for Local Staging of Prostate Cancer: A Diagnostic Meta-analysis. Eur Urol. 2016;70(2):233-45.
6. Eichelberg C, Erbersdobler A, Haese A, Schlomm T, Chun FK, Currlin E, et al. Frozen section for the management of intraoperatively detected palpable tumor lesions during nerve-sparing scheduled radical prostatectomy. Eur Urol. 2006;49(6):1011-6; discussion 6-8.
7. Beyer B, Schlomm T, Tennstedt P, Boehm K, Adam M, Schiffmann J, et al. A Feasible and Time-efficient Adaptation of NeuroSAFE for da Vinci Robot-assisted Radical Prostatectomy. Eur Urol. 2014;66(1):138-44.
8. Schlomm T, Tennstedt P, Huxhold C, Steuber T, Salomon G, Michl U, et al. Neurovascular structure-adjacent frozen-section examination (NeuroSAFE) increases nerve-sparing frequency and reduces positive surgical margins in open and robot-assisted laparoscopic radical prostatectomy: experience after 11,069 consecutive patients. Eur Urol. 2012;62(2):333-40.
9. Mirmilstein G, Rai BP, Gbolahan O, Srirangam V, Narula A, Agarwal S, et al. The neurovascular structure-adjacent frozen-section examination (NeuroSAFE) approach to nerve sparing in robot-assisted laparoscopic radical prostatectomy in a British setting - a prospective observational comparative study. BJU Int. 2018;121(6):854-62.
10. Fromont G, Baumert H, Cathelineau X, Rozet F, Validire P, Vallancien G. Intraoperative frozen section analysis during nerve sparing laparoscopic radical prostatectomy: feasibility study. J Urol. 2003;170(5):1843-6.
11. Kakiuchi Y, Choy B, Gordetsky J, Izumi K, Wu G, Rashid H, et al. Role of frozen section analysis of surgical margins during robot-assisted laparoscopic radical prostatectomy: a 2608-case experience. Hum Pathol. 2013;44(8):1556-62.
12. Lepor H, Kaci L. Role of intraoperative biopsies during radical retropubic prostatectomy. Urology. 2004;63(3):499-502.
13. Gillitzer R, Thuroff C, Fandel T, Thomas C, Thuroff JW, Brenner W, et al. Intraoperative peripheral frozen sections do not significantly affect prognosis

For peer review only

after nerve-sparing radical prostatectomy for prostate cancer. BJU Int. 2011;107(5):755-9.
14. Heinrich E, Schon G, Schiefelbein F, Michel MS, Trojan L. Clinical impact of intraoperative frozen sections during nerve-sparing radical prostatectomy. World J Urol. 2010;28(6):709-13.
15. Goharderakhshan RZ, Sudilovsky D, Carroll LA, Grossfeld GD, Marn R, Carroll PR. Utility of intraoperative frozen section analysis of surgical margins in region of neurovascular bundles at radical prostatectomy. Urology. 2002;59(5):709-14.
16. Shaw G RP, Sooriakumaran P, Briggs T, Nathan S, Ramachandran N, Freeman A, Brew-Graves C, Williams N, Adshead J, Vasdev N, Haese A, Kelly J. The NeuroSAFE PROOF study (An RCT to evaluate the use of froze section technology to improve oncological and functional outcomes in robotic radical prostatectomy). 2017. 2017;43(11):2213.
17. Elliott D, Husbands S, Hamdy FC, Holmberg L, Donovan JL. Understanding and Improving Recruitment to Randomised Controlled Trials: Qualitative Research Approaches. Eur Urol. 2017;72(5):789-98.
18. Chan AW, Tetzlaff JM, Gotzsche PC, Altman DG, Mann H, Berlin JA, et al. SPIRIT 2013 explanation and elaboration: guidance for protocols of clinical trials. BMJ. 2013;346:e7586.
19. Ficarra V, Novara G, Ahlering TE, Costello A, Eastham JA, Graefen M, et al. Systematic review and meta-analysis of studies reporting potency rates after robot-assisted radical prostatectomy. Eur Urol. 2012;62(3):418-30.
20. Sooriakumaran P, Srivastava A, Shariat SF, Stricker PD, Ahlering T, Eden CG, et al. A multinational, multi-institutional study comparing positive surgical margin rates among 22393 open, laparoscopic, and robot-assisted radical prostatectomy patients. Eur Urol. 2014;66(3):450-6.
21. Akin O RC, Ishill NM, Moskowitz CS, Zhang J, Hricak H. Interactive dedicated curriculum improves accuracy in the interpretation of MR imaging of prostate cancer. . European Radiology. 2010;20(4):995-1002.
22. Tewari AK, Srivastava A, Huang MW, Robinson BD, Shevchuk MM, Durand M, et al. Anatomical grades of nerve sparing: a risk-stratified approach to neural-hammock sparing during robot-assisted radical prostatectomy (RARP). BJU Int. 2011;108(6 Pt 2):984-92.
23. Vasdev N, Agarwal S, Rai BP, Soosainathan A, Shaw G, Chang S, et al. Intraoperative Frozen Section of the Prostate Reduces the Risk of Positive Margin Whilst Ensuring Nerve Sparing in Patients with Intermediate and High-Risk Prostate Cancer Undergoing Robotic Radical Prostatectomy: First Reported UK Series. Curr Urol. 2016;9(2):93-103.
24. Rosen RC RA, Wagner G, Osterloh IH, Kirkpatrick J, Mishra A. The international index of erectile function (IIEF): a multidimensional scale for assessment of erectile dysfunction. Urology. 1997;49(6):822-30.
25. Ware JE SC. The MOS 36-Item Short-Form Health Survey (SF-36): I. Conceptual Framework and Item Selection. Medical Care. 1992;30:473-83.
26. Avery K, Abrams P, Chadwick M, Donovan J. Development and psychometric evaluation of the ICIQ urinary and bowel incontinence modules: The ICIQ-UI and ICIQ-BI. Neurourol Urodynam. 2004;23(5-6):396-7.

For peer review only

27. Herdman M, Gudex C, Lloyd A, Janssen M, Kind P, Parkin D, et al. Development and preliminary testing of the new five-level version of EQ-5D (EQ-5D-5L). Qual Life Res. 2011;20(10):1727-36.
28. Dindo D, Demartines N, Clavien PA. Classification of surgical complications: a new proposal with evaluation in a cohort of 6336 patients and results of a survey. Ann Surg. 2004;240(2):205-13.

Appendices

1. Figure 1. Study Flow Chart.
2. Table 1. Table of Assessments.
3. Informed Consent Form.
4. Participant Information Sheet.

For peer review only

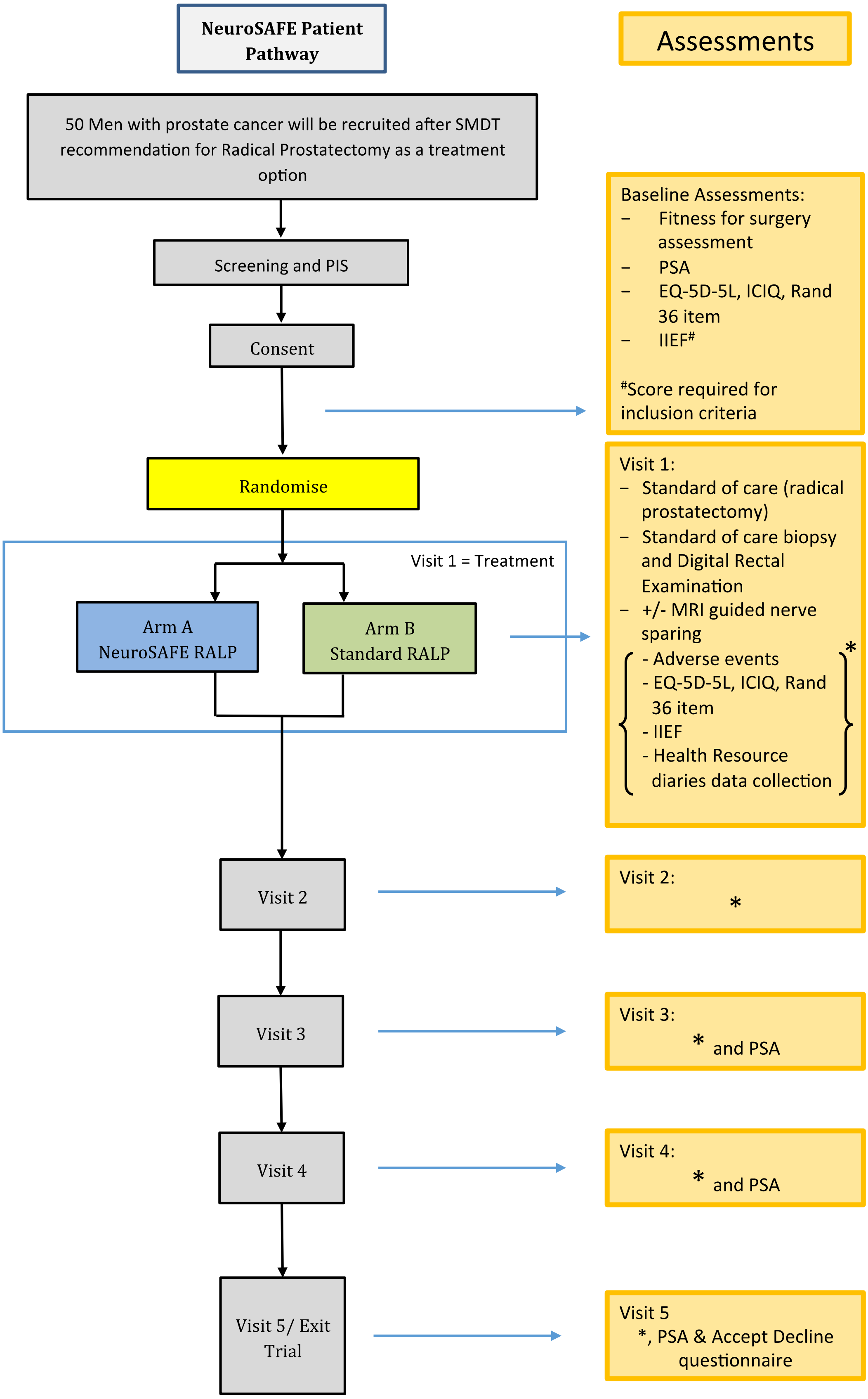
NeuroSAFE Patient Pathway
Assessments
50 Men with prostate cancer will be recruited after SMDT recommendation for Radical Prostatectomy as a treatment option
Screening and PIS
Consent
Baseline Assessments:
– Fitness for surgery assessment
– PSA
– EQ-5D-5L, ICIQ, Rand 36 item
– IIEF#
#Score required for inclusion criteria
Randomise
Visit 1 = Treatment
Arm A NeuroSAFE RALP
Arm B Standard RALP
Visit 1:
– Standard of care (radical prostatectomy)
– Standard of care biopsy and Digital Rectal Examination
– +/- MRI guided nerve sparing
- Adverse events
- EQ-5D-5L, ICIQ, Rand 36 item
- IIEF
- Health Resource diaries data collection
*
Visit 2
Visit 2: *
Visit 3
Visit 3: * and PSA
Visit 4
Visit 4: * and PSA
Visit 5/ Exit Trial
Visit 5 *, PSA & Accept Decline questionnaire

Table of Assessments

**Visit**	Screening/Baseline	Randomisation	Treatment Visit 1	Visit 2 6 weeks post op (+ 4 weeks)	Visit 3 3 months (±4 weeks)	Visit 4 6 months (±4 weeks)	Visit 5 12 months (±4 weeks)
Informed consent	x						
Randomisation		x					
Fitness for surgery assessment	x						
PSA	x				x	x	x
Standard care (radical prostatectomy)			x				
Standard care biopsy & DRE			x				
+/-MRI guided nerve sparing			x				
Adverse events			x	x	x	x	x
EQ-5D-5L, ICIQ, Rand 36 items, ICIQ	x		x	x	x	x	x
IIEF	X[1]		x	x	x	x	x
Health Resource Diaries data collection			x	x	x	x	x
Accept Decline Questionnaire							x

[1] needs to be done prior to randomisation as score is required for inclusion criteria, all other baseline questionnaires, need to be completed prior to telling the patient which treatment arm he has drawn.

For peer review only

For peer review only

Print on headed paper

(To be printed on hospital headed paper)

PARTICIPANT CONSENT FORM

A single blinded, multi-centre, feasibility study to evaluate the ability to randomise men with prostate cancer into a trial comparing NeuroSAFE Robotic assisted radical prostatectomy (RALP) to standard Robotic assisted radical prostatectomy (RALP)

Acronym: NeuroSAFE PROOF

Version: 2.0
Date: 15 FEB 2018

Subject Number:

Name of Researcher**:**

Please initial box

1. I confirm that I have read and understood the Patient Information Sheet [Version No. ______, dated _________] for the NeuroSAFE PROOF trial.

2. I understand that my participation is voluntary, and that I am free to withdraw at any time, without giving any reason, and understand that my medical care or legal rights will not be affected.

3. I understand that relevant sections of my medical notes and data collected during the study may be looked at by individuals from University College London and responsible persons authorised by the sponsor, from regulatory authorities or from the NHS Trust, where it is relevant to my taking part in this research.

4. I consent to the storage of personal information for the purposes of this study. This may include paper or electronic information. I understand that any information that could identify me will be kept strictly confidential and that no personal information will be included in the study report or other publications

5. I give permission for my GP to be informed of my inclusion in this study.

6. I give permission for my GP to be informed in the event of any unexpected significant findings.

For peer review only - http://bmjopen.bmj.com/site/about/guidelines.xhtml

For peer review only

7. I give permission for my full postal address to be recorded and stored. This will be used for research purposes only and will remain confidential. This information may be used for sending out quality of life questionnaires, if required. ☐

8. I agree to take part in the above study. ☐

....	....	....
**Name of participant**	**Date**	**Signature**
....	....	....
**Name of person taking consent**	**Date**	**Signature**

For peer review only

[TO BE PRINTED ON LOCAL HEADED PAPER]

PARTICIPANT INFORMATION SHEET

Long title A single blinded, multi-centre, feasibility study to evaluate the ability to randomise men with prostate cancer into a trial comparing NeuroSAFE Robotic assisted radical prostatectomy (RALP) to standard Robotic assisted radical prostatectomy (RALP).

Acronym: NeuroSAFE PROOF

Part A

PART A: SUMMARY PARTICIPANT INFORMATION SHEET

Your urologist has told you that you have prostate cancer and has recommended surgery to remove your bladder. This type of surgery is called radical prostatectomy.

We would like to invite you to take part in a study that will allow us to determine whether men are prepared to consider randomisation into a larger scale study comparing the benefits and disadvantages of treating patients like you with different types of radical prostatectomy. **50 men will take part in this study in the UK.**

If you decide to take part in the study, your research nurse or urologist will ask you to sign the consent form at the end of this information sheet. You will then be treated as follows:

- A computer will be used to decide which type of surgery you receive. The computer will use a process called randomisation to allocate at random half of the patients in the study to one type of surgery, and the other half to a different type of surgery.
- You will not need to attend the clinic any more often than normal practice for your condition. However, when you do attend we will carefully assess your recovery to help us to understand reasons, if any, why one type of prostate cancer surgery is more beneficial than another. This will happen at your hospital stay during and immediately after your surgery, and again at about four weeks and three months, after your surgery.

For peer review only - http://bmjopen.bmj.com/site/about/guidelines.xhtml

- You will be asked to complete Quality of Life questionnaires about your physical health and wellbeing. There will be 4 questionnaires, 5 at your last visit, to complete and we will ask you to do this at baseline, before you know which type of surgery you will have, at 6 weeks, 3, 6 and finally at 12 months after your surgery. In addition at the same time points you will be given health resource diaries to complete. At your last trial visit, 12 months post-surgery we will also ask you to complete an accept/decline questionnaire so we can find out a bit more about why you agreed to join the study.

- If you decide not to take part, your surgery will proceed in line with the standard of care.

- If after reading Part A: Summary Participant Information Sheet you are interested in participating in the study, please familiarise yourself with Part B: Detailed Participant Information Sheet on the following pages **before** signing the study consent form.

For peer review only

For peer review only - http://bmjopen.bmj.com/site/about/guidelines.xhtml

Part B

PART B: DETAILED PARTICIPANT INFORMATION SHEET

We would like to invite you to take part in this study. Before you decide whether to take part, you need to understand why the research is being done and what it would involve for you. Please take time to read the following information carefully. Ask us if there is anything that is not clear or if you would like more information. Take time to make your decision. You can talk to others (such as your GP, family and friends) about the study, if you wish, before reaching a decision.

1. What is the surgical procedure being tested?

What is a radical prostatectomy?

Radical prostatectomy is a complex surgical procedure to remove the prostate because of cancer. In this procedure damage to the nerves which run in the outer coverings of the prostate commonly causes erectile dysfunction (usually permanent) and urinary incontinence (usually temporary). The standard surgical approach involves careful planning of whether the nerves can be preserved (by carefully peeling them off the outside of the prostate during surgery) based on the results of the prostate biopsy and MRI scan along with an examination of the prostate at the start of the operation.

What is removed?

Your entire prostate and in some cases the lymph nodes (which drain the prostate) will also be removed.

What is NeuroSAFE?

NeuroSAFE is a technique developed in Germany to promote safe nerve sparing. This technique involves, during the operation, an examination of the prostate under a microscope by a pathologist to see whether prostate cancer is touching the nerves. If it is not, the nerve is left in place. If it is, the nerve and cancer cells are removed. This is the only difference between the NeuroSAFE technique and current standard care. We have performed 12 cases of NeuroSAFE at UCLH and achieved cancer control comparable to the standard operative procedure.

2. What are the side effects of the surgery?

Radical prostatectomy, with or without NeuroSAFE, is a complex surgical procedure with side effects and potential complications. Your urologist will have already given you a separate information sheet with details about what you can expect to happen, and the potential side effects and complications associated with radical prostatectomy.

For peer review only - http://bmjopen.bmj.com/site/about/guidelines.xhtml

What is the purpose of the study?

The NeuroSAFE procedure is designed to minimise side effects of the surgery without compromising cancer care. We plan to evaluate whether using NeuroSAFE is effective. This trial will evaluate whether men are prepared to be randomised between treatment groups, to see whether we can teach the procedure to a level of competence that randomisation is possible at other NHS centres. The ultimate objective will be to evaluate, through a large scale multicentre randomised controlled study, the effects of use of the NeuroSAFE procedure in terms of potency, urinary continence, quality of life as well as cancer control and the need for extra treatment with radiotherapy. A full evaluation of the cost effectiveness of the procedure (informing about value for money for the NHS) will be incorporated.

We hope this phase of the study will help us to find out:

- The proportion of men offered enrolment into the study who are willing to be randomised.
- The reasons why men might prefer not to participate.

All the participants in the study will receive radical prostatectomy with or without the NeuroSAFE procedure.

3. What are the possible benefits of taking part?

We hope that the surgery you receive will be an effective treatment for your cancer, although this cannot be guaranteed. If you take part, you may benefit from seeing the same research nurse at each of your assessment visits. You may also benefit from a more thorough review of your recovery after surgery. Information we get from this study may help us to treat patients with prostate cancers more effectively in the future.

4. What are the possible disadvantages and risks of taking part?

Treatment within this study is very similar to that you would receive if you were not participating. The inconvenience, complications, and impact on quality of life of the surgery you receive may vary dependent on the type of surgery you receive. Other disadvantages and/or risks of taking part might include:

- The inconvenience of completing questionnaires about how you feel. If you find that you are unable or unwilling to complete them for any reason, please discuss this with your local urology team.
- Your clinical assessments could result in the Research Doctor finding a condition of which you were unaware. Your GP will be informed with your consent so the appropriate medical treatment can be given to you.
- The only difference is that with NeuroSAFE, the prostate is examined under a microscope by a pathologist during the operation. Though it is unlikely, this may

For peer review only - http://bmjopen.bmj.com/site/about/guidelines.xhtml
For peer review only

add up to 15 minutes to the time the operation takes. This will not expose you to any increased risk.

5. Why have I been invited?

You have been invited to take part in this study because your urologist has found that you have a type of prostate cancer which is suitable for radical prostatectomy. About 50 men will take part in this study in the UK.

6. Do I have to take part?

No, It is up to you to decide whether or not to take part in this study. We will describe the study to you. We will go through this Participant Information Sheet with you and give you a copy to keep. If you do not wish to take part, you do not need to give a reason although if you were able to tell us why this would help us plan further study. If you do decide to take part, you will be asked to sign a consent form.

If you consent to take part in the trial, you are always free to withdraw at any time without giving a reason. If you decide not to take part, or to withdraw after consenting to take part, it will not affect the standard of care you receive. You will have the opportunity to discuss with your urologist the alternatives for treatment and/or surgery if you choose not to take part.

7. What will happen to me if I take part?

If you choose to take part in the study, your research nurse or urologist will ask you to sign the consent form.

Your research nurse and/or urologist will assess your medical condition and any recent investigations to make sure you are a suitable candidate, and then enter you into the trial. You will be allocated at random to either standard radical prostatectomy arm or the radical prostatectomy with NeuroSAFE arm.

As part of the normal care pathway, you will need to attend a pre-operative assessment clinic at the hospital as an out-patient at least 1-2 weeks before your surgery to assess your health.

On the day of your surgery you will be admitted and the operation will be performed. You will not be informed at any point as to whether you have had the NeuroSAFE procedure (this blinding to treatment allocation is an important measure to prevent bias within the results). You will have a post-surgery assessment and then again at about 6 weeks after your surgery, then at 3, 6 and 12 months. Before your 6 week appointment your prostate will be formally examined by the pathologist and this will be discussed at a committee. Whether extra treatment with radiotherapy is required will be considered by this committee and the outcome will be conveyed to you at the 6 week appointment. You will need to visit the hospital for a follow-up assessment. These follow up appointments will take place at the hospital you were treated at.

Preoperatively and at each postoperative visit you will be asked to complete 4 quality of life questionnaires about your physical health and wellbeing as part of the study. You will need to

For peer review only - http://bmjopen.bmj.com/site/about/guidelines.xhtml

complete these questionnaires during hospital assessment visits. You will also be given a health resource diary to assess your health care resource use, at these time points. We hope the questionnaires will help us to understand how your surgery has affected the quality of your life and out of pocket expenses. In addition, you will also be asked to complete an accept/decline questionnaire at your 12 month visit to help us understand why you agreed to take part in this trial. Your follow up appointments will be at 6 weeks, 3 months, 6 months and 12 months.

We will follow ethical and legal practice and all information about you will be handled in confidence. When you consent to join the study, you will be allocated a unique subject number, this is how all forms for the trial will be identified to protect your identity. This information will be held securely at your hospital on paper and electronically under the provisions of the 1998 Data Protection Act. Any information about you that leaves the hospital will have your name and address removed so that you cannot be recognised from it. All tissue removed during the operation will be held by the pathology department of the hospital, for as long as the hospital policy requires. Your GP will be notified of your participation in the study if you choose to take part. Should we by chance pick up any significant clinical findings we will inform both you and your GP.

8. What do I have to do?

You will need to visit the hospital for your surgery and assessments, as described above. All of these assessments are normally recommended for patients who undergo radical prostatectomy and will not involve additional visits to your hospital.

It may be possible for you to participate in other research studies during your treatment but you should discuss this with your research nurse or urologist first. Please tell your research nurse or urologist if you have had any other treatments recently as they might make you unsuitable for this study.

9. What are the alternatives for treatment?

If you decide not to take part, your treatment will proceed in line with standard practice at your hospital.

10. What happens when the research study stops?

When all the participants have been entered into the study, we will review the success of the study. When all the participants have completed the study, we will compare how participants have responded to the two types of surgery, and assess how participants' quality of life, recovery and out of pocket expenses have been affected by the surgery they have received.

11. What if something goes wrong?

Any complaint about the conduct of the trial, the way you have been dealt with during the study, or any possible harm you might suffer, will be addressed. If something does go wrong and you are harmed during the research due to someone's negligence, then you may have grounds for

For peer review only - http://bmjopen.bmj.com/site/about/guidelines.xhtml

a legal action for compensation against the organisations involved, including the sponsor (University College London), and the National Health Service (NHS) Trust. However, you may have to pay your legal costs. The NHS national complaints mechanisms will still be available to you (if appropriate). If you remain unhappy and wish to complain formally, you can do this through the Patient advice and liaison services (PALS) team.

PALS
Site to include local details

Telephone: site to include local phone number

Will my taking part in this study be kept confidential?

Yes. We will follow ethical and legal practice and all information about you will be handled in confidence. Your GP will be notified of your participation into the study with your consent if you choose to take part.

With your consent your medical records may be looked at by people who monitor and audit trials, UK regulatory authorities and representatives from the sponsor's office, to check that the study is being done properly. All will have a duty of confidentiality to you as a research participant.

12. What if new information becomes available?

Sometimes during the course of a research project, new information becomes available which may affect you. If this happens, your urologist will tell you about it and discuss with you whether you want to continue in the study. If you decide to withdraw, your urologist will make arrangements for your care to continue. If you decide to continue in the study, you may be asked to sign an updated consent form. On receiving new information your urologist might consider it to be in your best interests to withdraw you from the study. Your urologist will explain the reasons and arrange for your care to continue.

13. What will happen if I don't want to carry on with the study?

You may withdraw from either the study surgery or from the entire study at any time. If you withdraw from surgery, we will ask your consent to keep in contact with us to let us follow-up your progress. Information collected may then still be used. Alternatively, you can withdraw from the entire study with no effect on your standard care, any information collected prior to your withdrawal of consent will be maintained, no further data will be collected once you withdraw consent.

14. What will happen to the results of the research study?

The results of this study may be shown at medical meetings and submitted to major urology and cancer research journals for publication. You will not be identified in any way in any

For peer review only - http://bmjopen.bmj.com/site/about/guidelines.xhtml

report or publication arising from the study. If you would like copies of publications please let a member of the research team know.

If you wish to know the results at the end of the study, please contact your urologist or research nurse.

15. Who is organising and funding the research?

The chief investigator for the trial is Mr Greg L Shaw, based at University College London Hospital, UK. The study is funded by the UCH charitable trustees, the Rosetrees Trust, and the National Institute for Health Research for Patient Benefit. University College London is acting as the sponsor for the study.

16. Who has reviewed the study?

All research in the NHS is looked at by an independent group of people, called a research ethics committee to protect your safety, rights, well-being and dignity. This study has been reviewed by the London - Central Research Ethics Committee and by the sponsor's office at UCL.

<u>Contact for further information</u>:

If you have any further questions concerning this study please contact your site research team:

Principal Investigator (**urologist**):

Name: …………………………………… **on** ……………………………………

Or your **research/specialist nurse**:

Name: …………………………………… **on** ……………………………………

<u>Who else can I talk to?</u>

Alternatively, if you or your relatives have any questions about this study you may wish to contact the following organisation that is independent of the hospital at which you are being treated:

Macmillan Cancer Support is a registered charity providing information about all aspects of cancer for patients and their families. They can provide useful booklets on bladder cancer, the treatments for bladder cancer and medical research in general. You may contact their specialist cancer nurses on **0808 800 1234**. You can also access their web site at **www. http://www.macmillan.org.uk**.

<u>What to do next</u>

For peer review only - http://bmjopen.bmj.com/site/about/guidelines.xhtml

If you are at all unsure whether to take part in this study, you can have more time to think it over.

You will be given a copy of this information sheet and the signed consent form to keep.

Thank you for taking the time to consider participating in the study and for reading this leaflet.

Chief Investigator: Mr Greg L. Shaw (Honorary Senior Lecturer)
UCL Division of Surgery & Interventional Science.
Charles Bell House, 3rd floor, W1W 7JN
Tel: +44 (0)20 7679 6490
Fax: +44 (0)20 7679 6470

Sponsor Organisation: University College London

Favourable ethics opinion granted by London - Central Research Ethics Committee.

For peer review only - http://bmjopen.bmj.com/site/about/guidelines.xhtml

Reporting checklist for protocol of a clinical trial.

Based on the SPIRIT guidelines.

Instructions to authors

Complete this checklist by entering the page numbers from your manuscript where readers will find each of the items listed below.

Your article may not currently address all the items on the checklist. Please modify your text to include the missing information. If you are certain that an item does not apply, please write "n/a" and provide a short explanation.

Upload your completed checklist as an extra file when you submit to a journal.

In your methods section, say that you used the SPIRIT reporting guidelines, and cite them as:

Chan A-W, Tetzlaff JM, Altman DG, Laupacis A, Gøtzsche PC, Krleža-Jerić K, Hróbjartsson A, Mann H, Dickersin K, Berlin J, Doré C, Parulekar W, Summerskill W, Groves T, Schulz K, Sox H, Rockhold FW, Rennie D, Moher D. SPIRIT 2013 Statement: Defining standard protocol items for clinical trials. Ann Intern Med. 2013;158(3):200-207

		Reporting Item	Page Number
Title	#1	Descriptive title identifying the study design, population, interventions, and, if applicable, trial acronym	1
Trial registration	#2a	Trial identifier and registry name. If not yet registered, name of intended registry	2
Trial registration: data set	#2b	All items from the World Health Organization Trial Registration Data Set	4
Protocol version	#3	Date and version identifier	15
Funding	#4	Sources and types of financial, material, and other support	14
Roles and responsibilities: contributorship	#5a	Names, affiliations, and roles of protocol contributors	1
Roles and responsibilities:	#5b	Name and contact information for the trial sponsor	1

For peer review only

sponsor contact information			
Roles and responsibilities: sponsor and funder	#5c	Role of study sponsor and funders, if any, in study design; collection, management, analysis, and interpretation of data; writing of the report; and the decision to submit the report for publication, including whether they will have ultimate authority over any of these activities	14
Roles and responsibilities: committees	#5d	Composition, roles, and responsibilities of the coordinating centre, steering committee, endpoint adjudication committee, data management team, and other individuals or groups overseeing the trial, if applicable (see Item 21a for data monitoring committee)	13
Background and rationale	#6a	Description of research question and justification for undertaking the trial, including summary of relevant studies (published and unpublished) examining benefits and harms for each intervention	3
Background and rationale: choice of comparators	#6b	Explanation for choice of comparators	3
Objectives	#7	Specific objectives or hypotheses	4
Trial design	#8	Description of trial design including type of trial (eg, parallel group, crossover, factorial, single group), allocation ratio, and framework (eg, superiority, equivalence, non-inferiority, exploratory)	5
Study setting	#9	Description of study settings (eg, community clinic, academic hospital) and list of countries where data will be collected. Reference to where list of study sites can be obtained	6
Eligibility criteria	#10	Inclusion and exclusion criteria for participants. If applicable, eligibility criteria for study centres and individuals who will perform the interventions (eg, surgeons, psychotherapists)	6
Interventions: description	#11a	Interventions for each group with sufficient detail to allow replication, including how and when they will be administered	9

For peer review only

Interventions: modifications	#11b	Criteria for discontinuing or modifying allocated interventions for a given trial participant (eg, drug dose change in response to harms, participant request, or improving / worsening disease)	9
Interventions: adherance	#11c	Strategies to improve adherence to intervention protocols, and any procedures for monitoring adherence (eg, drug tablet return; laboratory tests)	9
Interventions: concomitant care	#11d	Relevant concomitant care and interventions that are permitted or prohibited during the trial	9
Outcomes	#12	Primary, secondary, and other outcomes, including the specific measurement variable (eg, systolic blood pressure), analysis metric (eg, change from baseline, final value, time to event), method of aggregation (eg, median, proportion), and time point for each outcome. Explanation of the clinical relevance of chosen efficacy and harm outcomes is strongly recommended	11
Participant timeline	#13	Time schedule of enrolment, interventions (including any run-ins and washouts), assessments, and visits for participants. A schematic diagram is highly recommended (see Figure)	7
Sample size	#14	Estimated number of participants needed to achieve study objectives and how it was determined, including clinical and statistical assumptions supporting any sample size calculations	6
Recruitment	#15	Strategies for achieving adequate participant enrolment to reach target sample size	8
Allocation: sequence generation	#16a	Method of generating the allocation sequence (eg, computer-generated random numbers), and list of any factors for stratification. To reduce predictability of a random sequence, details of any planned restriction (eg, blocking) should be provided in a separate document that is unavailable to those who enrol participants or assign interventions	8
Allocation concealment	#16b	Mechanism of implementing the allocation sequence (eg, central telephone; sequentially numbered, opaque, sealed	9

For peer review only

mechanism		envelopes), describing any steps to conceal the sequence until interventions are assigned	
Allocation: implementation	#16c	Who will generate the allocation sequence, who will enrol participants, and who will assign participants to interventions	8
Blinding (masking)	#17a	Who will be blinded after assignment to interventions (eg, trial participants, care providers, outcome assessors, data analysts), and how	5
Blinding (masking): emergency unblinding	#17b	If blinded, circumstances under which unblinding is permissible, and procedure for revealing a participant's allocated intervention during the trial	5
Data collection plan	#18a	Plans for assessment and collection of outcome, baseline, and other trial data, including any related processes to promote data quality (eg, duplicate measurements, training of assessors) and a description of study instruments (eg, questionnaires, laboratory tests) along with their reliability and validity, if known. Reference to where data collection forms can be found, if not in the protocol	10
Data collection plan: retention	#18b	Plans to promote participant retention and complete follow-up, including list of any outcome data to be collected for participants who discontinue or deviate from intervention protocols	10
Data management	#19	Plans for data entry, coding, security, and storage, including any related processes to promote data quality (eg, double data entry; range checks for data values). Reference to where details of data management procedures can be found, if not in the protocol	12
Statistics: outcomes	#20a	Statistical methods for analysing primary and secondary outcomes. Reference to where other details of the statistical analysis plan can be found, if not in the protocol	11
Statistics: additional analyses	#20b	Methods for any additional analyses (eg, subgroup and adjusted analyses)	11
Statistics: analysis population and missing data	#20c	Definition of analysis population relating to protocol non-adherence (eg, as randomised analysis), and any statistical methods to handle missing data (eg, multiple imputation)	11

For peer review only

Data monitoring: formal committee	#21a	Composition of data monitoring committee (DMC); summary of its role and reporting structure; statement of whether it is independent from the sponsor and competing interests; and reference to where further details about its charter can be found, if not in the protocol. Alternatively, an explanation of why a DMC is not needed	12
Data monitoring: interim analysis	#21b	Description of any interim analyses and stopping guidelines, including who will have access to these interim results and make the final decision to terminate the trial	11
Harms	#22	Plans for collecting, assessing, reporting, and managing solicited and spontaneously reported adverse events and other unintended effects of trial interventions or trial conduct	12
Auditing	#23	Frequency and procedures for auditing trial conduct, if any, and whether the process will be independent from investigators and the sponsor	12
Research ethics approval	#24	Plans for seeking research ethics committee / institutional review board (REC / IRB) approval	13
Protocol amendments	#25	Plans for communicating important protocol modifications (eg, changes to eligibility criteria, outcomes, analyses) to relevant parties (eg, investigators, REC / IRBs, trial participants, trial registries, journals, regulators)	14
Consent or assent	#26a	Who will obtain informed consent or assent from potential trial participants or authorised surrogates, and how (see Item 32)	8
Consent or assent: ancillary studies	#26b	Additional consent provisions for collection and use of participant data and biological specimens in ancillary studies, if applicable	n/a
Confidentiality	#27	How personal information about potential and enrolled participants will be collected, shared, and maintained in order to protect confidentiality before, during, and after the trial	12
Declaration of interests	#28	Financial and other competing interests for principal investigators for the overall trial and each study site	14
Data access	#29	Statement of who will have access to the final trial dataset,	12

For peer review only

		and disclosure of contractual agreements that limit such access for investigators	
Ancillary and post trial care	#30	Provisions, if any, for ancillary and post-trial care, and for compensation to those who suffer harm from trial participation	12
Dissemination policy: trial results	#31a	Plans for investigators and sponsor to communicate trial results to participants, healthcare professionals, the public, and other relevant groups (eg, via publication, reporting in results databases, or other data sharing arrangements), including any publication restrictions	13
Dissemination policy: authorship	#31b	Authorship eligibility guidelines and any intended use of professional writers	13
Dissemination policy: reproducible research	#31c	Plans, if any, for granting public access to the full protocol, participant-level dataset, and statistical code	13
Informed consent materials	#32	Model consent form and other related documentation given to participants and authorised surrogates	17
Biological specimens	#33	Plans for collection, laboratory evaluation, and storage of biological specimens for genetic or molecular analysis in the current trial and for future use in ancillary studies, if applicable	n/a

The SPIRIT checklist is distributed under the terms of the Creative Commons Attribution License CC-BY-ND 3.0. This checklist was completed on 13. November 2018 using http://www.goodreports.org/, a tool made by the EQUATOR Network in collaboration with Penelope.ai

For peer review only

BMJ Open

NeuroSAFE Robot Assisted Laparoscopic Prostatectomy versus standard Robot Assited Laparoscopic Prostatectomy for men with localized prostate cancer (NeuroSAFE PROOF): protocol for a randomised controlled trial feasibility study.

Journal:	*BMJ Open*
Manuscript ID	bmjopen-2018-028132.R1
Article Type:	Protocol
Date Submitted by the Author:	11-Apr-2019
Complete List of Authors:	Dinneen, Eoin; University College Hospital London, Department of Urology<br>Haider, Aiman; University College Hospital London<br>Allen, Clare; University College Hospital London, Department of Radiology<br>Freeman, Alex; University College Hospital London, Department of Histopathology<br>Briggs, Tim; University College Hospital London, Department of Urology<br>Nathan, Senthil; University College Hospital London, Department of Urology<br>Brew-Graves, Chris; Division of Surgery and Interventional Science, University College London<br>Grierson, Jack; University College London Medical School, Division of Surgery & Interventional Science;<br>Williams, Norman; University College London, UCL Division of Surgery & Interventional Science<br>Persad, Raj; North Bristol NHS Trust<br>Oakley, Neil; Sheffield Teaching Hospitals NHS Foundation Trust<br>Adshead, Jim; Lister Hospital, Department of Urology<br>Huland, Hartwig; Universitatsklinikum Hamburg Eppendorf Universitares Herzzentrum Hamburg GmbH, Department of Urology<br>Haese, Alexander; Universitatsklinikum Hamburg Eppendorf Universitares Herzzentrum Hamburg GmbH, Department of Urology<br>Shaw, Greg; University College Hospital London, Department of Urology
**Primary Subject Heading**:	Urology
Secondary Subject Heading:	Surgery
Keywords:	NeuroSAFE, nerve sparing, frozen section, robotic prostatectomy, prostate cancer

For peer review only - http://bmjopen.bmj.com/site/about/guidelines.xhtml

NeuroSAFE Robot Assisted Laparoscopic Prostatectomy versus standard Robot Assisted Laparoscopic Prostatectomy for men with localized prostate cancer (NeuroSAFE PROOF): protocol for a randomised controlled trial feasibility study.

Eoin Dinneen[1], Aiman Haider[2], Clare Allen[1], Alex Freeman[2], Tim Briggs[1], Senthil Nathan[1], Chris Brew-Graves[3], Jack Grierson[3], Norman R Williams[3], Raj Persad[4], Neil Oakley[5], Jim Adshead[6], Hartwig Huland[7], Alexander Haese[7], Greg Shaw[1].

Author Affiliations

1. University College Hospital London, Department of Urology, Westmoreland Street Hospital, 16-18 Westmoreland Street, London, W1G 8PH.
2. University College Hospital London, Department of Histopathology, 235 Euston Road, London NW1 2BU.
3. Surgical & Interventional Trials Unit, University College London, Charles Bell House, 43-45 Foley Street, London, W1W 7JN.
4. North Bristol Hospitals Trust, Department of Urology, Southmead Hospital, Southmead Lane, Westbury-on-Trym, Bristol, BS10 5NB.
5. Sheffield Teaching Hospitals NHS Trust, Department of Urology, Royal Hallamshire Hospital, Glossop Road, S10 2JF.
6. East and North Hertfordshire NHS Trust, Department of Urology, The Lister Hospital, Coreys Mill Lane, Stevenage, SG1 4AB.
7. Martini Klinik, Department of Urology, University Hospital Eppendorf, Martini-strasse 52, 20246 Hamburg.

Corresponding Author

Mr Eoin Dinneen BA (Hons), MRCS.
University College Hospital London, Department of Urology, Westmoreland Street Hospital, 16-18 Westmoreland Street, London, W1G 8PH.

eoindinneen@gmail.com

Word Count: 4,912.

Author email addresses (in the order they appear on authorship list):

aiman.haider@nhs.net, clare.allen@uclh.nhs.uk, alex.freeman2@nhs.net, tim.briggs@nhs.net, s.nathan@nhs.net, c.brew-graves@ucl.ac.uk, j.grierson@ucl.ac.uk, norman.williams@ucl.ac.uk, Raj.Persad@nbt.nhs.uk, neiloakley@btinternet.com, j.adshead@nhs.net, h.huland@uke.de, haese@uke.de, gregshaw@nhs.net.

Keywords

For peer review only - http://bmjopen.bmj.com/site/about/guidelines.xhtml

NeuroSAFE, nerve sparing, frozen section, potency, robotic prostatectomy, prostate cancer.

Abstract

Introduction

Robot assisted laparoscopic prostatectomy (RALP) offers potential cure for localized prostate cancer, but is associated with considerable toxicity. Potency and urinary continence are improved when the neurovascular bundles (NVB) are spared during a nerve spare (NS) RALP. There is reluctance, however, to perform NS RALP when there are concerns that the cancer extends beyond the capsule of the prostate into the NVB, as NS RALP in this instance increases the risk of a positive surgical margin (PSM). The NeuroSAFE technique involves intraoperative fresh frozen section analysis of the postero-lateral aspect of the prostate margin to assess whether cancer extends beyond the capsule. There is evidence from large observational studies that functional outcomes can be improved and PSM rates reduced when the NeuroSAFE technique is used during RALP. To date, however, there has been no randomised controlled trial (RCT) to substantiate this finding. The NeuroSAFE PROOF feasibility study is designed to assess whether it is feasible to randomise men to NeuroSAFE RALP versus a control arm of 'standard of practice' RALP.

Methods

NeuroSAFE PROOF feasibility study will be a multicentre, single blinded RCT with patients randomised 1:1 to either NeuroSAFE RALP (intervention) or standard RALP (control). Treatment allocation will occur after trial entry and consent. The primary outcome will be assessed as the successful accrual of 50 men at 3 sites over 15 months. Secondary outcomes will be used to aid subsequent power calculations for the definitive full-scale RCT and will include rates of NS, PSM, biochemical recurrence, adjuvant treatments , and patient reported functional outcomes on potency, continence and quality of life.

Ethics and dissemination

NeuroSAFE PROOF has ethical approval (REC reference 17/LO/1978). NeuroSAFE PROOF is supported NIHR Research for Patient Benefit funding (NIHR reference PB-PG-1216-20013). Findings will be made available through peer-reviewed publications.

Trial Registration number

NCT03317990

For peer review only

For peer review only - http://bmjopen.bmj.com/site/about/guidelines.xhtml

Strengths and Limitations of this study

- This is the first feasibility clinical trial to compare NeuroSAFE RALP to a UK 'standard of care' RALP.
- Multicentre, randomised controlled trial design.
- This is the protocol for a feasibility study, and therefore this study is not powered to allow for the analysis of secondary outcomes.
- Secondary outcomes include validated patient reported outcome questionnaires, histological and oncological endpoints, and health economics.

Introduction

Nerve sparing (NS) robot-assisted laparoscopic radical prostatectomy (RALP) is associated with superior post-operative functional outcomes such as erectile function and possibly urinary continence. (1, 2) While functional results after RP are of importance to many men, the primary objective of an cancer operation remains complete eradication of the tumour.(3) Therefore, it is important that performing NS RALP does not compromise that oncologic outcome. Positive surgical margins (PSM) are associated with greater risk of biochemical recurrence (4), adjuvant therapies (which negate any improved functional outcomes following NS RALP), and disease progression. As such, despite the improved anatomical understanding and technological advancement of the robotic platform, NS RALP is often eschewed in favour of assuring the safety of a negative surgical margin by performing wide excision around the prostate. Uncertainty in this area is compounded by the fact that the accuracy of pre-operative imaging techniques and physical examination to detect extra-capsular extension (ECE) and/or neurovascular cancer involvement are debatable.(5) In particular, pooled data from a recent diagnostic meta-analysis found MRI to have a limited sensitivity of 0.57(95% Confidence Interval 0.49-0.64) when predicting ECE.(6) Therefore, RALP can often lead to unwarranted sacrificing of important functioning neurovascular bundles (NVB). When deciding whether to perform NS RALP or non-NS RALP, surgeons rely on parameters such as pre-operative erectile function, D'Amico Risk Classification, radiological staging, and location and volume of tumour to cautiously assess the safety of an NS approach. These assessments may not give a true picture and are prone to subjective evaluation. The concept of a frozen section-navigated NS during RALP using neurovascular structure adjacent frozen section examination of the prostate resection margin (NeuroSAFE) has been described by the Martini-Clinik in Hamburg, Germany. (5, 7, 8) These authors and others report benefit in functional outcomes and improved oncologic safety in their series (9, 10) though other retrospective series are not as clear-cut.(11)

The NeuroSAFE technique has not yet been widely adopted, as concerns remain that it is time and resource consuming, has low sensitivity and specificity, and has potentially conflicting oncologic results.(12-15) Neither intraoperative fresh frozen section (FFS) in RALP nor the NeuroSAFE technique have been prospectively evaluated by a randomised controlled trial (RCT). Moreover, few studies have assessed the impact of FFS during RALP on longer term patient

For peer review only - http://bmjopen.bmj.com/site/about/guidelines.xhtml

outcomes such as biochemical recurrence, adjuvant cancer treatments (such as radiotherapy and hormones), and comprehensive functional outcomes.

Research need

To determine whether the NeuroSAFE technique (fresh frozen section of the prostate tissue adjacent to the neurovascular bundles) during RALP is helpful to surgical teams (and therefore patients) who are balancing the competing goals of cancer control and functional optimization. An attempt to answer this question will require a multi-dimensional approach focusing on pre-operative and operative parameters, final histological outcomes, adjuvant treatments, quality of life, erectile function, urinary continence and health economics. There is recognition that surgical RCTs can be hard to recruit to and that patients may not accept their allocated treatment option.(16) For this reason we propose to undertake a feasibility study to examine recruitment rates, acceptance of allocated treatment and to rehearse collection of outcomes.

Study aims and outcomes

The aim is to prospectively recruit for randomisation eligible patients to either standard RALP (control arm) or NeuroSAFE RALP (intervention arm). This feasibility trial has a single blinded, 1:1 randomised design. This article reports the protocol (v.2.0, 6 February 2018) for the NeuroSAFE PROOF trial and follows SPIRIT (Standard Protocol Items: Recommendations for Interventional Trials) reporting guidelines.(17)

The trial objectives are to assess the feasibility and acceptability of:

- Recruiting men with localized prostate cancer to an RCT of NeuroSAFE RALP versus standard RALP .
- Collectiong data for outcome measures, including patient reported outcomes.
- Estimating treatment effects in order to inform power calculations for the definitive full-scale future trial.
- The study's procedures, interventions and follow-up regimen amongst men being treated with RALP for localized prostate cancer.

The following criteria will have to be met to proceed to a full-scale trial:

- Recruitment of 50 men over 15 months from opening. Fifty men was decided upon in order to demonstrate that if similar recruitment rates were maintained in the full-scale NeuroSAFE PROOF study, the trial would be able to recruit the several hundreds of men likely necessary to

For peer review only - http://bmjopen.bmj.com/site/about/guidelines.xhtml

appropriately power the said trial over the course of approximately 2-3 years.

- Recruitment and performance of procedures (both intervention and control) as per allocation at 3 pre-specified participating sites (UCLH, Bristol and Sheffield). At least 2 treatments (one intervention, one control) should be performed at each site.

- Methodological or practical issues with the trial design should be identified and amended before full-scale trial.

- Good acceptability of the intervention among patients and their families, indicated in qualitative feedback and Public & Patient Involvement events.

- Acquisition of comprehensive patient reported outcomes measure including health economics questionnaires.

Public and Patient Involvement (PPI)

Patient feedback on the design of the study was obtained at two NeuroSAFE PROOF PPI sessions on 12 July 2018 and 20th September 2018. The second event was attended by men participating in NeuroSAFE PROOF. The PPI events were supported by Macmillan Cancer (Charity no. 261017) and Orchid (Charity no. 1080540). Participants, patients and their families were asked specifically about the level of blinding, the burden of follow-up appointments, and priorities in their recovery from RALP. Following their feedback, NeuroSAFE PROOF now informs men following surgery of their NS status, though blinding to allocation status (intervention or control) is maintained. Furthermore, men expressed keen preference to know their treatment allocation once exiting the 12 months follow-up period, and this is now incorporated into trial design. Patient representatives sit on the trial steering committee for NeuroSAFE PROOF and share oversight of the management of the trial. The study is also funded by National Institute for Healthcare Research Research for Patient Benefit stream, which has patient members on their decision panels. On completion of NeuroSAFE PROOF, prostate cancer patient groups will be consulted again on amendments to the design of the full-scale RCT. The results will be published following peer review, and anonymised data will be presented at national and international conferences.

Methods and Analysis

Trial Design

NeuroSAFE PROOF feasibility study is a prospective, multicentre, feasibility RCT in patients undergoing RALP for localised prostate cancer. Eligible patients will be consented and randomised 1:1 to NeuroSAFE RALP (intervention) or

standard RALP (control) after multidisciplinary team (MDT) review in National Health Service (NHS) urological cancer centres. It is not possible to blind the surgical team to the treatment received on the day of surgery. Researchers for whom knowledge of allocation is imperative, ie those involved in operating on patients or coordinating operating lists or pathology teams are not blinded to treatment allocation, other members of the research team are blinded to treatment allocation. Participants are not informed of treatment allocation until completing 12 months follow-up and exiting the study, though they are informed of their ultimate nerve-spare status (i.e. no nerve spare, unilateral nerve spare, bilateral nerve spare). The primary outcome is feasibility of recruitment.

Secondary outcomes will include:

- Rates of NS performed during RALP.
- Rates of positive surgical margins.
- Adjuvant therapies and Biochemical Recurrence.
- Patient reported outcome questionnaires assessing potency, urinary continence, and quality of life.
- Patient reported health care resource diaries.

These outcome measures will allow us to explore the feasibility and acceptability of delivering a full-scale multicentre RCT. The decision to include the 50 feasibility study patients in the full-scale NeuroSAFE PROOF trial will only be allowed if the feasibility study aligns sufficiently closely, and will be at the discretion of the independent trial steering committee (TSC).

Trial Population

Prior to entry, patients must be accurately staged (e.g. mpMRI prostate and cross-sectional imaging to assess for distant metastases (e.g. bone scan or whole body MRI)), within 3 months prior to randomization. Eligible patients must have had their case discussed at NHS cancer MDT and deemed suitable and fit for RALP. Eligible participants will fulfil all the inclusion criteria and none of the exclusion criteria as defined below:

Inclusion Criteria

i. Men opting to undergo RALP for organ confined prostate cancer.
ii. Potent men (IIEF 22-25 not using PDE51 or other medications or vacuum pump)
iii. Men who are continent of urine (no self-reported urinary incontinence)
iv. Able to give written informed consent to participate.

Exclusion Criteria

i. Unable to undergo RALP
ii. Known overactive bladder
iii. Previous treatment for prostate cancer

For peer review only - http://bmjopen.bmj.com/site/about/guidelines.xhtml

iv. Previous/current hormone treatment for prostate cancer
v. Nerve sparing deemed futile due to locally advanced disease by surgeon and radiologist.

An overview of the study schema can be seen in Figure 1.

Sample Size

The primary outcome of NeuroSAFE PROOF is to demonstrate adequate recruitment to prove feasibility of the full-scale definitive NeuroSAFE PROOF RCT. Operative data, preliminary functional outcomes data, and preliminary oncological outcomes data from this feasibility data will be used to help determine power calculations for the full-scale NeuroSAFE PROOF RCT. Previous literature suggests that 80% of men undergoing bilateral NS will have erections sufficient for penetrative sex, 40% of men undergoing unilateral NS and 10% of men undergoing no NS. (18)

Recruitment

NeuroSAFE PROOF will recruit patients attending NHS cancer centres. All patients who have a diagnosis of prostate cancer and who have been recommended for RALP by a specialist NHS regional multi-disciplinary team meeting will be eligible to be approached.

Consent

Written informed consent will be obtained from each patient prior to study entry and performing baseline trial assessments. An ethics committee approved patient information sheet will be provided to facilitate this process. Prospective participants will be given at least a week to read the patient information sheet prior to being re-approached with regards to recruitment. The investigator, or their designee, must ensure adequate explanations of the trial, that participation is voluntary, and they can withdraw at any time. In consenting to the trial participants understand that they are consenting to provide study follow-up and data collection. A patient may withdraw from the trial at any time without prejudice to his subsequent treatment.

Randomisation

Patients will be randomised using an online system (https://www.sealedenvelope.com/trials/) on a 1:1 basis to either NeuroSAFE RALP or standard RALP. A computer-generated adaptive minimization algorithm that incorporates a random element will be used to ensure treatment groups are balanced (stratified) for centre. Treatment allocation will occur after trial entry and consent. Participants will not be informed of their treatment allocation until exiting the trial 12 months following their surgery. The clinical teams performing and coordinating surgery will not be blinded to treatment

For peer review only

allocation as this is impractical, any members of the research team not involved in these activities will be blinded.

Setting

Participants will be recruited from NHS cancer centres undertaking RALP who have the ability to perform the additional NeuroSAFE technique. Recruiting sites will be invited by the Trial Management Group (TMG). Trial sites will have well-developed RALP programs; routinely performing at least 250 cases per year and undergoing satisfactory NHS quality assurance and safety visits.

Surgeon and unit accreditation

Variations in surgical team performance can produce differences in outcomes from RALP.(19) As such, to minimize this potential source of confounding, surgeons and surgical teams participating in NeuroSAFE PROOF feasibility study will require accreditation from the TMG. Further, surgeons performing trial treatment need to have completed more than 100 cases and have submitted these data to the BAUS Oncology database.

Robot-assisted Laparoscopic Prostatectomy (RALP)

Patients will undergo RALP using the DaVinci surgical system as is standard of care in the NHS. All patients will undergo a pre-operative multi-parametric MRI (mpMRI) that will be interpreted by a consultant genitourinary radiologist with at least 2 years experience in reading prostate mpMRIs. The pre-operative mpMRI will be interpreted by the radiologist with biopsy information and will be used to evaluate presence of cancer and likelihood of extra-capsular extension in zones according to the PIRADS anatomic division of the prostate at the base, the mid gland and the apex. In each zone, using a 1-5 scale (1, definitely absent; 2, probably absent; 3, possibly present; 4, probably present; 5 definitely present), they will record the likelihood of tumour on each side. Using the same 1-5 scale they then indicated the likelihood of extra-capsular extension in each corresponding zone as has been previously done by Akin et al.(20) Subsequently, the radiologist using the mpMRI makes a NS recommendation for each side of the prostate for each participant regardless of treatment arm allocation. The radiological NS recommendation will be recorded:

- Nerve Sparing: Yes
- Nerve Sparing: No
- Digital rectal examination dependent.

Control Arm: Standard RALP

Standard RALP (control arm) is performed as per NHS routine practice. Pre-operative parameters used to guide surgeon NS decision include mpMRI review with genitourinary radiologist recommendation with regards NS, prostate biopsy histology, and digital rectal examination under general anaesthesia. Individual

For peer review only - http://bmjopen.bmj.com/site/about/guidelines.xhtml

surgeons are asked after RALP to grade the quality of NS performed on each side numerically as seen below as previously described (21):

- Grade 4 - No nerve spare. Wide excision of lateral pelvic fascia (LPF) and Denonvilliers' fascia.
- Grade 3 - Limited nerve spare, or partial/incremental nerve spare. Incision through outer compartment of LPF.
- Grade 2 – Interfascial nerve spare. LPF is taken just outside the layer of the veins of the prostate capsule. Still largely preserving the large neural trunks (also known as the NVBs).
- Grade 1 – Intrafascial nerve spare. LPF is taken just outside the prostate capsule. Represents greatest possible NS.

Detailed times of starting the RALP and finishing the RALP are recorded on the day of surgery in order to calculate the length of each case.

Intervention Arm: NeuroSAFE RALP

NeuroSAFE RALP (intervention arm) will be performed in accordance with previously described methods, initially developed at the Martini Clinik, Hamburg, Germany.(5, 8, 22) The additional steps outlined include nerve sparing technique and apical dissection, specimen removal, intra-operative frozen section protocol, simultaneous urethra-vesical anastomosis (+/- pelvic lymphadenectomy where performed), pathological processing of specimen, pathology reporting protocol, and secondary excision of the neurovascular bundle (where appropriate). Detailed results of the frozen section examination will be collected and included in the results, including number of sections positive, length of positive margin, identity and grade of pathologist. When the frozen section examination demonstrates cancer at the margin of the prostate as per pathology reporting protocol, secondary excision of the NVB is described by the surgeon in one of three ways: 1. No tissue resected, 2. Local excision of Denonvilliers'/peri-prostatic fascia, or 3. Entire bundle resected. Secondarily resected tissue (after fresh frozen section pathology phone call, when performed) is sent for routine paraffin embedded histological analysis and is not analysed as part of the intraoperative fresh frozen section. Detailed times of the beginning of the RALP, the removal of the prostate for specimen painting, arrival of specimen in laboratory, communication of details of fresh frozen section to the surgical team, and finishing the RALP are recorded on the day of surgery.

Participating sites all visited the central site (UCLH) prior to their Site Initiation Visits in order to receive teaching and standardisation in the surgical and histopathological aspects of NeuroSAFE RALP (intervention arm). Subsequently, researchers from the central site (GS and AH) reciprocated the visit for the first NeuroSAFE RALP performed by each site to ensure fidelity to technique protocol.

Data Collection

Post-treatment trial Assessments will be conducted at follow-up appointments. All patients will have follow-up appointments at 6 weeks following surgery, 3

For peer review only - http://bmjopen.bmj.com/site/about/guidelines.xhtml

months, 6 months and finally 12 months following their treatment. The Table of Assessments is demonstrated below (Table 1):

Table 1. Table of Assessments

	Baseline/ Recruitment	Visit 1 Treatment	Visit 2 (6 weeks post-RALP)	Visit 3 (3 months)	Visit 4 (6 months)	Visit 5 (12 months)
Informed consent	x					
Randomisation	x					
PSA	x			x	x	x
Standard RALP or NeuroSAFE RALP		x				
Adverse events		x	x			
EQ-5D-5L, ICIQ, Rand 36	x		x	x	x	x
IIEF	x		x	x	x	x
Adjuvant Therapies			x	x	x	x
Health Resource Diary			x	x	x	x

Time points:

i. Baseline/preoperative: at time of consent, trial entry and randomisation to treatment allocation.
ii. Visit 1. Operative parameters recorded and any immediate post-operative complications/adverse events.
iii. Outpatient follow-up. Visits 2, 3, 4 and 5 will record patient reported outcomes measures and health care resource diaries. Adjuvant treatments and oncological outcomes will be recorded prospectively alongside functional assessments.
iv. On Visits 3, 4 and 5 a serum PSA will be taken in addition to functional questionnaires and adjuvant treatment outcomes.

Secondary endpoint measures include

i. IIEF-15 (baseline, 6 weeks, 3 months, 6 months and 12 months): a self-completion tool for men focusing on erectile function and sex life. Measured domains include erectile function, orgasmic function, sexual desire, intercourse satisfaction and overall satisfaction.(23)
ii. Rand-36-item Health Survey (baseline, 6 weeks, 3 months, 6 months and 12 months): a self-completion questionnaire that laps eight concepts: physical functioning, bodily pain, role limitations due to health problems, role limitations due to personal or emotional problems, emotional well-being, social functioning, energy/fatigue, and general health perceptions.(24)
iii. International Consultation on Incontinence Questionnaire (ICIQ) (baseline, 6 weeks, 3 months, 6 months, and 12 months): a self-

For peer review only

completion tool for patients to subjectively measure frequency and severity of urinary loss, and impact on quality of life for those with urinary incontinence.(25)

i. EQ-5D-5L (baseline, 6 weeks, 3 months, 6 months, and 12 months): a self-completion tool for patients that is applicable to a wide range of health conditions and treatments. Measured domains include mobility, self-care, usual activities, pain and anxiety or depression.(26)

ii. Health resource diaries (6 weeks, 3 months, 6 months, six month visit diary will be returned at the 12 month visit),. This will allow the collection of resource use data from point of operation untill trial exit at 12 months. These diaries are non-validated.

iii. Post-operative: adverse events and complications will be recorded. Clavien-Dindo classification of surgical complications will be used to assess for any surgical complications as per normal hospital practice.

iv. Histology: following RALP the following details will be recorded as per standard histological analysis of prostatectomy mount: histological type, Gleason Grade, Gleason group, tumour volume, extra prostatic extension, seminal vesicle involvement, lymphovascular invasion, description of margin involvement (including apical, basal, circumferential), tumour stage, nodes, positive surgical margins.

v. Oncological outcomes (3 months, 6 months and 12 months): the curative outcomes from RALP will be examined to determine local and distant recurrence, metastases, PSA and biochemical recurrence, need for adjuvant therapies and survival (overall and cancer specific).

Statistical Analysis

As NeuroSAFE PROOF is a feasibility trial, there is no intention to undertake detailed statistical analysis. Preliminary analysis will be performed after 5 cases have reached 'Visit 3' to rehearse data extraction, completeness of follow-up, fidelity of data, and by proxy acceptability of follow-up measures. Further preliminary data analysis, maintaining blinding, of the secondary outcomes 'margin status' and 'RALP nerve spare status' will be performed by the data monitoring committee (DMC) after 40 surgeries have been performed to evaluate and help revise power estimations for the full-scale RCT. Potential bias due to missing data will be investigated by comparing descriptively the baseline characteristics of the trial participants with complete outcome measurements to those who have missing outcome measurements. Men will be offered the option of telephone follow-up and/or be sent questionnaires by post if they are unable to attend clinic appointments for follow-up. Additionally, patients wishing to withdraw from the trial will be counselled regarding end of active participation, as this will allow the trial team to continue to use their outcome data for an intention to treat (ITT) analysis. Records will be kept of all participants allocated to a treatment arm who do not undergo allocated treatment with explanatory notes. These instances will be highlighted to the surgical & interventional trial unit at University College London (study sponsor) (SITU) and the trial steering committee for judgment on whether inclusion in outcomes is appropriate.

For peer review only - http://bmjopen.bmj.com/site/about/guidelines.xhtml

Safety

The number of adverse events related to serious adverse events (SAEs) will be summarised descriptively by arm, by grade and body system. RALP is a major surgery that has a number of recognized complications and a very low risk of death (less than 1 in 100). Operative/post-operative RALP complications will be graded using the Clavien-Dindo classification. The central trial management team will ask sites to submit complication data blinded by arm of treatment. This will be assigned Clavien-Dindo classification centrally.(27) All SAEs will be recorded in the medical records, the CRF, the sponsor's adverse event log, and an SAE form. The site principal investigator (PI) or designated individual will complete an SAE form, and the form will be sent to SITU within five working days of becoming aware of the event. The study chief investigator or site PI will respond to any SAE queries raised by the sponsor as soon as possible. Where the event is unexpected and thought to be related to the procedure, this must be reported by the PI to SITU, who will then inform the Health Research Authority within 15 days.

Data Monitoring

This trial will use an electronic case report form (eCRF), and trial data will be entered into an approved, protected database (https://neurosafe.slms.ucl.ac.uk). Access to the eCRF system will only be provided to staff with the appropriate authority. Participants will be given a unique number and subject identifier. Data will be entered under this identification number onto the central database stored on the servers. The database will be password protected and only accessible to members of the NeuroSAFE study team as well as external regulators if requested. The servers are protected by firewalls and are patched and maintained according to best practice. The physical location of the servers is protected by CCTV and security door access. The database software provides a number of features to help maintain data quality, including: maintaining an audit trail, allowing custom validations on all data, allowing users to raise data query requests and search facilities to identify validation failure/missing data. After completion of the study, the database will be retained on the servers of University College London for on-going analysis of secondary outcomes. The identification, screening and enrolment logs, linking participant identifiable data to the pseudo-anonymised subject numbers will be held in written form in a locked filing cabinet. After completion of the study, sites will store screening and enrolment logs securely for 10 years.

Trial Funding, organization and administration

The trial was developed by the NeuroSAFE PROOF trial management group (TMG) and has been funded by University College London Hospitals NHS Foundation Trust, The Rosetrees Foundation, and the NIHR Research for Patient Benefit (RfPB) stream (reference: PB-PG-1216-200113). Applied Medical are contributing disposable laparoscopic trocar ports suitable for use in NeuroSAFE

For peer review only - http://bmjopen.bmj.com/site/about/guidelines.xhtml

RALP (intervention arm), but the company has had no role in trial design and will have no role in trial implementation, analysis, interpretation or writing any reports. The trial is sponsored by UCL and has registered sponsor reference number 17/0443 and ClinicalTrials.gov (NCT03317990) on 23 October 2017 with an amendment made on 1 June 2018. All members of the trial are Good Clinical Practice trained. A Data Monitoring Committee (DMC) will monitor patient safety and the rate of recruitment of subjects in the study. They will meet at least once a year while the trial is ongoing for routine review of safety data and trial progression. They have power to call additional meetings and review data at any point in the trial should they wish to do so. The DMC may report their findings to the TSC. The TSC is an independent committee consisting of relevant, experienced clinicians and researchers. The TSC will ensure the study is conforming to governance requirements as set out by the trial sponsor. The TSC will meet at least once a year. The sponsor may also arrange an independent trial monitor to review the study data.

Ethics and Dissemination

Ethical approval for NeuroSAFE PROOF was granted on 6th February 2018 (REC reference 17/LO/1978). Here, we report version 2.0 of the protocol. The sponsors, HRA, and REC will approve any future amendments as appropriate. Similarly, all participating sites have (or will have) gained local regional ethics committee (REC) prior to receiving a site initiation visit and being given the permission to open recruitment.

Non-blinded results of the study will be published in peer-reviewed publications and will be presented at relevant national and international conferences. The TMG will not present the arms in comparison to one another to avoid loss of equipoise and introduction of bias into the full-scale RCT. The TMG will work with a patient panel to develop lay reports to disseminate research findings to patient groups and the clinical teams at participating sites.

Discussion

Intra-operative FFS analysis of the NVB adjacent prostate margin during RALP to guide NS is now an established technique in a number of centres. Published large series from these centres demonstrate improvements on their outcomes, both functional and oncological. In spite of the possible benefit to with localized prostate cancer undergoing surgery, the NeuroSAFE technique during RALP has not been widely introduced in the UK. The lack of Level 1 evidence to support NeuroSAFE RALP is a valid reason for this.

The NeuroSAFE PROOF RCT feasibility study will be the first trial to assess the feasibility of conducting a randomised trial to evaluate intraoperative frozen section evaluation of the prostate margin during RALP anywhere in the world. The results of this feasibility trial will be used to prepare the full-scale NeuroSAFE PROOF RCT.

Trial Status

For peer review only - http://bmjopen.bmj.com/site/about/guidelines.xhtml

NeuroSAFE PROOF RCT feasibility study opened to recruitment in April 2018 using protocol version 2.0 (6 February 2018) and is due to close to recruitment in January 2020 or after the 50th patient is consented and randomised. NeuroSAFE PROOF RCT feasibility study will therefore close in January 2021 or when the last participant to undergo treatment completes the 12 month follow-up as per protocol. Amendments were reviewed and approved by the sponsor and the Regional Ethics Committee. Protocol amendments are disseminated to relevant parties by SITU.

Acknowledgements

We gratefully thank the participants, PIs, research nurses, all the clinicians involved in providing care, regional cancer service coordinators, data managers and other site staff who have been responsible for setting up, recruiting participants and collecting the data for the trial. We are grateful for the trial oversight provided by the sponsor and the members of the TSC. The TSC members are Jack Cuzick (Chair), Queen Mary's University of London, Alastair Lamb, Oxford, Imran Ahmad, Glasgow, and Abay Mulatu, Coventry.

Contributors

Conception and design of NeuroSAFE PROOF trial: GS, ED, AH, JG, AF, TB, SN, CA, JA, HH, A. Haese, NW, RP, NO and CB-G. Writing of the manuscript: ED, AH, CA, AF, RP, NO, CB-G, JG, and GS. All authors have read and approved the final manuscript. The trial will comply with the authorship criteria recommended but the International Committee of Medical Journal Editors.

Funding

The trial was funded by the NIHR RfPB and The Rosetrees Foundation. ED is funded by the NIHR RfPB.

Disclaimer

The funder had no role in the design, analysis, or collection of the data; in writing the manuscript; or in the decision to submit the manuscript for publication.

Competing Interests

Within NeuroSAFE PROOF, laparoscopic ports are supplied by Applied Medical but Applied Medical has had no role in the design, analysis, or collection of the data; in writing the manuscript; or in the decision to submit the manuscript for publication.

For peer review only - http://bmjopen.bmj.com/site/about/guidelines.xhtml

References

1. Steineck G, Bjartell A, Hugosson J, Axen E, Carlsson S, Stranne J, et al. Degree of preservation of the neurovascular bundles during radical prostatectomy and urinary continence 1 year after surgery. Eur Urol. 2015;67(3):559-68.
2. Savera AT, Kaul S, Badani K, Stark AT, Shah NL, Menon M. Robotic radical prostatectomy with the "Veil of Aphrodite" technique: histologic evidence of enhanced nerve sparing. Eur Urol. 2006;49(6):1065-73; discussion 73-4.
3. Ficarra V, Sooriakumaran P, Novara G, Schatloff O, Briganti A, Van der Poel H, et al. Systematic review of methods for reporting combined outcomes after radical prostatectomy and proposal of a novel system: the survival, continence, and potency (SCP) classification. Eur Urol. 2012;61(3):541-8.
4. Sooriakumaran P, Ploumidis A, Nyberg T, Olsson M, Akre O, Haendler L, et al. The impact of length and location of positive margins in predicting biochemical recurrence after robot-assisted radical prostatectomy with a minimum follow-up of 5 years. BJU Int. 2015;115(1):106-13.
5. Eichelberg C, Erbersdobler A, Haese A, Schlomm T, Chun FK, Currlin E, et al. Frozen section for the management of intraoperatively detected palpable tumor lesions during nerve-sparing scheduled radical prostatectomy. Eur Urol. 2006;49(6):1011-6; discussion 6-8.
6. de Rooij M, Hamoen EH, Witjes JA, Barentsz JO, Rovers MM. Accuracy of Magnetic Resonance Imaging for Local Staging of Prostate Cancer: A Diagnostic Meta-analysis. Eur Urol. 2016;70(2):233-45.
7. Beyer B, Schlomm T, Tennstedt P, Boehm K, Adam M, Schiffmann J, et al. A Feasible and Time-efficient Adaptation of NeuroSAFE for da Vinci Robot-assisted Radical Prostatectomy. Eur Urol. 2014;66(1):138-44.
8. Schlomm T, Tennstedt P, Huxhold C, Steuber T, Salomon G, Michl U, et al. Neurovascular structure-adjacent frozen-section examination (NeuroSAFE) increases nerve-sparing frequency and reduces positive surgical margins in open and robot-assisted laparoscopic radical prostatectomy: experience after 11,069 consecutive patients. Eur Urol. 2012;62(2):333-40.

For peer review only - http://bmjopen.bmj.com/site/about/guidelines.xhtml

9. Mirmilstein G, Rai BP, Gbolahan O, Srirangam V, Narula A, Agarwal S, et al. The neurovascular structure-adjacent frozen-section examination (NeuroSAFE) approach to nerve sparing in robot-assisted laparoscopic radical prostatectomy in a British setting - a prospective observational comparative study. BJU Int. 2018;121(6):854-62.
10. Fromont G, Baumert H, Cathelineau X, Rozet F, Validire P, Vallancien G. Intraoperative frozen section analysis during nerve sparing laparoscopic radical prostatectomy: feasibility study. J Urol. 2003;170(5):1843-6.
11. Kakiuchi Y, Choy B, Gordetsky J, Izumi K, Wu G, Rashid H, et al. Role of frozen section analysis of surgical margins during robot-assisted laparoscopic radical prostatectomy: a 2608-case experience. Hum Pathol. 2013;44(8):1556-62.
12. Lepor H, Kaci L. Role of intraoperative biopsies during radical retropubic prostatectomy. Urology. 2004;63(3):499-502.
13. Gillitzer R, Thuroff C, Fandel T, Thomas C, Thuroff JW, Brenner W, et al. Intraoperative peripheral frozen sections do not significantly affect prognosis after nerve-sparing radical prostatectomy for prostate cancer. BJU Int. 2011;107(5):755-9.
14. Heinrich E, Schon G, Schiefelbein F, Michel MS, Trojan L. Clinical impact of intraoperative frozen sections during nerve-sparing radical prostatectomy. World J Urol. 2010;28(6):709-13.
15. Goharderakhshan RZ, Sudilovsky D, Carroll LA, Grossfeld GD, Marn R, Carroll PR. Utility of intraoperative frozen section analysis of surgical margins in region of neurovascular bundles at radical prostatectomy. Urology. 2002;59(5):709-14.
16. Elliott D, Husbands S, Hamdy FC, Holmberg L, Donovan JL. Understanding and Improving Recruitment to Randomised Controlled Trials: Qualitative Research Approaches. Eur Urol. 2017;72(5):789-98.
17. Chan AW, Tetzlaff JM, Gotzsche PC, Altman DG, Mann H, Berlin JA, et al. SPIRIT 2013 explanation and elaboration: guidance for protocols of clinical trials. BMJ. 2013;346:e7586.
18. Ficarra V, Novara G, Ahlering TE, Costello A, Eastham JA, Graefen M, et al. Systematic review and meta-analysis of studies reporting potency rates after robot-assisted radical prostatectomy. Eur Urol. 2012;62(3):418-30.
19. Sooriakumaran P, Srivastava A, Shariat SF, Stricker PD, Ahlering T, Eden CG, et al. A multinational, multi-institutional study comparing positive surgical margin rates among 22393 open, laparoscopic, and robot-assisted radical prostatectomy patients. Eur Urol. 2014;66(3):450-6.
20. Akin O RC, Ishill NM, Moskowitz CS, Zhang J, Hricak H. Interactive dedicated curriculum improves accuracy in the interpretation of MR imaging of prostate cancer. . European Radiology. 2010;20(4):995-1002.
21. Tewari AK, Srivastava A, Huang MW, Robinson BD, Shevchuk MM, Durand M, et al. Anatomical grades of nerve sparing: a risk-stratified approach to neural-hammock sparing during robot-assisted radical prostatectomy (RARP). BJU Int. 2011;108(6 Pt 2):984-92.
22. Vasdev N, Agarwal S, Rai BP, Soosainathan A, Shaw G, Chang S, et al. Intraoperative Frozen Section of the Prostate Reduces the Risk of Positive Margin Whilst Ensuring Nerve Sparing in Patients with Intermediate and High-

For peer review only - http://bmjopen.bmj.com/site/about/guidelines.xhtml

Risk Prostate Cancer Undergoing Robotic Radical Prostatectomy: First Reported UK Series. Curr Urol. 2016;9(2):93-103.
23. Rosen RC RA, Wagner G, Osterloh IH, Kirkpatrick J, Mishra A. The international index of erectile function (IIEF): a multidimensional scale for assessment of erectile dysfunction. Urology. 1997;49(6):822-30.
24. Ware JE SC. The MOS 36-Item Short-Form Health Survey (SF-36): I. Conceptual Framework and Item Selection. Medical Care. 1992;30:473-83.
25. Avery K, Abrams P, Chadwick M, Donovan J. Development and psychometric evaluation of the ICIQ urinary and bowel incontinence modules: The ICIQ-UI and ICIQ-BI. Neurourol Urodynam. 2004;23(5-6):396-7.
26. Herdman M, Gudex C, Lloyd A, Janssen M, Kind P, Parkin D, et al. Development and preliminary testing of the new five-level version of EQ-5D (EQ-5D-5L). Qual Life Res. 2011;20(10):1727-36.
27. Dindo D, Demartines N, Clavien PA. Classification of surgical complications: a new proposal with evaluation in a cohort of 6336 patients and results of a survey. Ann Surg. 2004;240(2):205-13.

Figure Legend

1. Figure 1. NeuroSAFE PROOF Feasibility Study Schema.

For peer review only

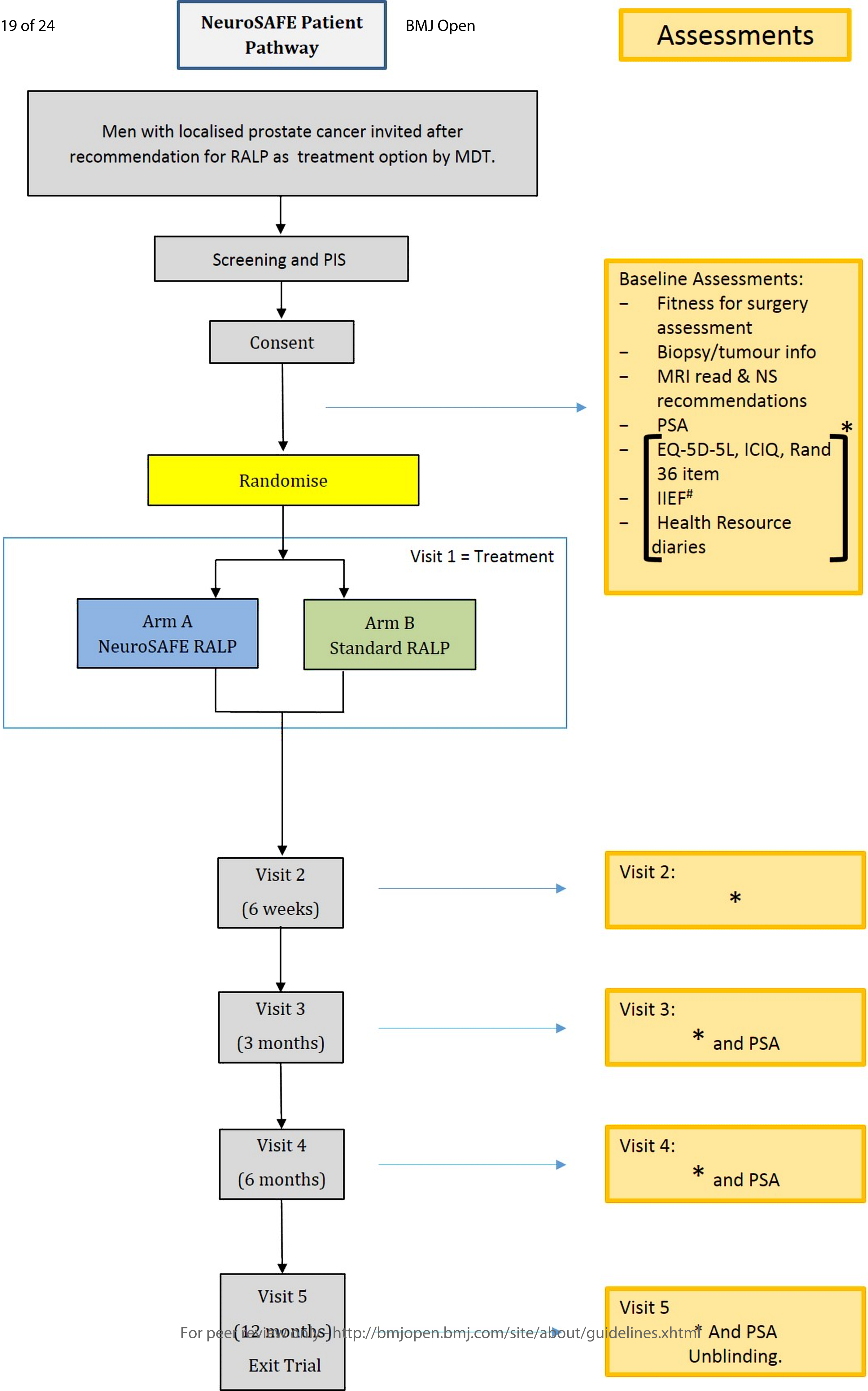

For peer review only - http://bmjopen.bmj.com/site/about/guidelines.xhtml

Reporting checklist for protocol of a clinical trial.

Based on the SPIRIT guidelines.

Instructions to authors

Complete this checklist by entering the page numbers from your manuscript where readers will find each of the items listed below.

Your article may not currently address all the items on the checklist. Please modify your text to include the missing information. If you are certain that an item does not apply, please write "n/a" and provide a short explanation.

Upload your completed checklist as an extra file when you submit to a journal.

In your methods section, say that you used the SPIRIT reporting guidelines, and cite them as:

Chan A-W, Tetzlaff JM, Altman DG, Laupacis A, Gøtzsche PC, Krleža-Jerić K, Hróbjartsson A, Mann H, Dickersin K, Berlin J, Doré C, Parulekar W, Summerskill W, Groves T, Schulz K, Sox H, Rockhold FW, Rennie D, Moher D. SPIRIT 2013 Statement: Defining standard protocol items for clinical trials. Ann Intern Med. 2013;158(3):200-207

		Reporting Item	Page Number
Title	#1	Descriptive title identifying the study design, population, interventions, and, if applicable, trial acronym	1
Trial registration	#2a	Trial identifier and registry name. If not yet registered, name of intended registry	2
Trial registration: data set	#2b	All items from the World Health Organization Trial Registration Data Set	4
Protocol version	#3	Date and version identifier	15
Funding	#4	Sources and types of financial, material, and other support	14
Roles and responsibilities: contributorship	#5a	Names, affiliations, and roles of protocol contributors	1
Roles and responsibilities:	#5b	Name and contact information for the trial sponsor	1

For peer review only

sponsor contact information			
Roles and responsibilities: sponsor and funder	#5c	Role of study sponsor and funders, if any, in study design; collection, management, analysis, and interpretation of data; writing of the report; and the decision to submit the report for publication, including whether they will have ultimate authority over any of these activities	14
Roles and responsibilities: committees	#5d	Composition, roles, and responsibilities of the coordinating centre, steering committee, endpoint adjudication committee, data management team, and other individuals or groups overseeing the trial, if applicable (see Item 21a for data monitoring committee)	13
Background and rationale	#6a	Description of research question and justification for undertaking the trial, including summary of relevant studies (published and unpublished) examining benefits and harms for each intervention	3
Background and rationale: choice of comparators	#6b	Explanation for choice of comparators	3
Objectives	#7	Specific objectives or hypotheses	4
Trial design	#8	Description of trial design including type of trial (eg, parallel group, crossover, factorial, single group), allocation ratio, and framework (eg, superiority, equivalence, non-inferiority, exploratory)	5
Study setting	#9	Description of study settings (eg, community clinic, academic hospital) and list of countries where data will be collected. Reference to where list of study sites can be obtained	6
Eligibility criteria	#10	Inclusion and exclusion criteria for participants. If applicable, eligibility criteria for study centres and individuals who will perform the interventions (eg, surgeons, psychotherapists)	6
Interventions: description	#11a	Interventions for each group with sufficient detail to allow replication, including how and when they will be administered	9

For peer review only

Interventions: modifications	#11b	Criteria for discontinuing or modifying allocated interventions for a given trial participant (eg, drug dose change in response to harms, participant request, or improving / worsening disease)	9
Interventions: adherance	#11c	Strategies to improve adherence to intervention protocols, and any procedures for monitoring adherence (eg, drug tablet return; laboratory tests)	9
Interventions: concomitant care	#11d	Relevant concomitant care and interventions that are permitted or prohibited during the trial	9
Outcomes	#12	Primary, secondary, and other outcomes, including the specific measurement variable (eg, systolic blood pressure), analysis metric (eg, change from baseline, final value, time to event), method of aggregation (eg, median, proportion), and time point for each outcome. Explanation of the clinical relevance of chosen efficacy and harm outcomes is strongly recommended	11
Participant timeline	#13	Time schedule of enrolment, interventions (including any run-ins and washouts), assessments, and visits for participants. A schematic diagram is highly recommended (see Figure)	7
Sample size	#14	Estimated number of participants needed to achieve study objectives and how it was determined, including clinical and statistical assumptions supporting any sample size calculations	6
Recruitment	#15	Strategies for achieving adequate participant enrolment to reach target sample size	8
Allocation: sequence generation	#16a	Method of generating the allocation sequence (eg, computer-generated random numbers), and list of any factors for stratification. To reduce predictability of a random sequence, details of any planned restriction (eg, blocking) should be provided in a separate document that is unavailable to those who enrol participants or assign interventions	8
Allocation concealment	#16b	Mechanism of implementing the allocation sequence (eg, central telephone; sequentially numbered, opaque, sealed	9

For peer review only

mechanism		envelopes), describing any steps to conceal the sequence until interventions are assigned	
Allocation: implementation	#16c	Who will generate the allocation sequence, who will enrol participants, and who will assign participants to interventions	8
Blinding (masking)	#17a	Who will be blinded after assignment to interventions (eg, trial participants, care providers, outcome assessors, data analysts), and how	5
Blinding (masking): emergency unblinding	#17b	If blinded, circumstances under which unblinding is permissible, and procedure for revealing a participant's allocated intervention during the trial	5
Data collection plan	#18a	Plans for assessment and collection of outcome, baseline, and other trial data, including any related processes to promote data quality (eg, duplicate measurements, training of assessors) and a description of study instruments (eg, questionnaires, laboratory tests) along with their reliability and validity, if known. Reference to where data collection forms can be found, if not in the protocol	10
Data collection plan: retention	#18b	Plans to promote participant retention and complete follow-up, including list of any outcome data to be collected for participants who discontinue or deviate from intervention protocols	10
Data management	#19	Plans for data entry, coding, security, and storage, including any related processes to promote data quality (eg, double data entry; range checks for data values). Reference to where details of data management procedures can be found, if not in the protocol	12
Statistics: outcomes	#20a	Statistical methods for analysing primary and secondary outcomes. Reference to where other details of the statistical analysis plan can be found, if not in the protocol	11
Statistics: additional analyses	#20b	Methods for any additional analyses (eg, subgroup and adjusted analyses)	11
Statistics: analysis population and missing data	#20c	Definition of analysis population relating to protocol non-adherence (eg, as randomised analysis), and any statistical methods to handle missing data (eg, multiple imputation)	11

For peer review only

Data monitoring: formal committee	#21a	Composition of data monitoring committee (DMC); summary of its role and reporting structure; statement of whether it is independent from the sponsor and competing interests; and reference to where further details about its charter can be found, if not in the protocol. Alternatively, an explanation of why a DMC is not needed	12
Data monitoring: interim analysis	#21b	Description of any interim analyses and stopping guidelines, including who will have access to these interim results and make the final decision to terminate the trial	11
Harms	#22	Plans for collecting, assessing, reporting, and managing solicited and spontaneously reported adverse events and other unintended effects of trial interventions or trial conduct	12
Auditing	#23	Frequency and procedures for auditing trial conduct, if any, and whether the process will be independent from investigators and the sponsor	12
Research ethics approval	#24	Plans for seeking research ethics committee / institutional review board (REC / IRB) approval	13
Protocol amendments	#25	Plans for communicating important protocol modifications (eg, changes to eligibility criteria, outcomes, analyses) to relevant parties (eg, investigators, REC / IRBs, trial participants, trial registries, journals, regulators)	14
Consent or assent	#26a	Who will obtain informed consent or assent from potential trial participants or authorised surrogates, and how (see Item 32)	8
Consent or assent: ancillary studies	#26b	Additional consent provisions for collection and use of participant data and biological specimens in ancillary studies, if applicable	n/a
Confidentiality	#27	How personal information about potential and enrolled participants will be collected, shared, and maintained in order to protect confidentiality before, during, and after the trial	12
Declaration of interests	#28	Financial and other competing interests for principal investigators for the overall trial and each study site	14
Data access	#29	Statement of who will have access to the final trial dataset,	12

For peer review only

		and disclosure of contractual agreements that limit such access for investigators	
Ancillary and post trial care	#30	Provisions, if any, for ancillary and post-trial care, and for compensation to those who suffer harm from trial participation	12
Dissemination policy: trial results	#31a	Plans for investigators and sponsor to communicate trial results to participants, healthcare professionals, the public, and other relevant groups (eg, via publication, reporting in results databases, or other data sharing arrangements), including any publication restrictions	13
Dissemination policy: authorship	#31b	Authorship eligibility guidelines and any intended use of professional writers	13
Dissemination policy: reproducible research	#31c	Plans, if any, for granting public access to the full protocol, participant-level dataset, and statistical code	13
Informed consent materials	#32	Model consent form and other related documentation given to participants and authorised surrogates	17
Biological specimens	#33	Plans for collection, laboratory evaluation, and storage of biological specimens for genetic or molecular analysis in the current trial and for future use in ancillary studies, if applicable	n/a

The SPIRIT checklist is distributed under the terms of the Creative Commons Attribution License CC-BY-ND 3.0. This checklist was completed on 13. November 2018 using http://www.goodreports.org/, a tool made by the EQUATOR Network in collaboration with Penelope.ai

For peer review only
